# PIK3CA mutations in androgen receptor-positive triple negative breast cancer confer sensitivity to the combination of PI3K and androgen receptor inhibitors

**DOI:** 10.1186/s13058-014-0406-x

**Published:** 2014-08-08

**Authors:** Brian D Lehmann, Joshua A Bauer, Johanna M Schafer, Christopher S Pendleton, Luojia Tang, Kimberly C Johnson, Xi Chen, Justin M Balko, Henry Gómez, Carlos L Arteaga, Gordon B Mills, Melinda E Sanders, Jennifer A Pietenpol

**Affiliations:** 1 Department of Biochemistry, Vanderbilt-Ingram Cancer Center, Vanderbilt University School of Medicine, Preston Research Building, 2200 Pierce Avenue, Nashville, 37232 TN USA; 2 Department of Biostatistics, Vanderbilt-Ingram Cancer Center, Vanderbilt University School of Medicine, Preston Research Building, 2200 Pierce Avenue, Nashville, 37232 TN USA; 3 Department of Medicine, Vanderbilt-Ingram Cancer Center, Vanderbilt University School of Medicine, Preston Research Building, 2200 Pierce Avenue, Nashville, 37232 TN USA; 4 Instituto Nacional de Enfermedades Neoplásicas, Av. Angamos Este 2520, Surquillo, Lima 34 Peru; 5 Department of Systems Biology, The University of Texas MD Anderson Cancer Center, 1400 Pressler Street, Unit 1484, Houston, 77030 TX USA; 6 Department of Pathology Vanderbilt-Ingram Cancer Center, Vanderbilt University School of Medicine, Preston Research Building, 2200 Pierce Avenue, Nashville, 37232 TN USA; 7 Vanderbilt-Ingram Cancer Center, 652 Preston Research Building, Nashville, 37232 TN USA

## Abstract

**Introduction:**

Triple negative breast cancer (TNBC) is a heterogeneous collection of biologically diverse cancers, which contributes to variable clinical outcomes. Previously, we identified a TNBC subtype that has a luminal phenotype and expresses the androgen receptor (AR+). TNBC cells derived from these luminal AR + tumors have high frequency phosphatidylinositol-4,5-bisphosphate 3-kinase catalytic subunit alpha (PIK3CA) mutations. The purpose of this study was to determine if targeting phosphoinositide 3-kinase (PI3K) alone or in combination with an AR antagonist is effective in AR + TNBC.

**Methods:**

We determined the frequency of activating PIK3CA mutations in AR + and AR- TNBC clinical cases. Using AR + TNBC cell line and xenograft models we evaluated the effectiveness of PI3K inhibitors, used alone or in combination with an AR antagonist, on tumor cell growth and viability.

**Results:**

PIK3CA kinase mutations were highly clonal, more frequent in AR + vs. AR- TNBC (40% vs. 4%), and often associated with concurrent amplification of the PIK3CA locus. PI3K/mTOR inhibitors had an additive growth inhibitory effect when combined with genetic or pharmacological AR targeting in AR + TNBC cells. We also analyzed the combination of bicalutamide +/- the pan-PI3K inhibitor GDC-0941 or the dual PI3K/mTOR inhibitor GDC-0980 in xenograft tumor studies and observed additive effects.

**Conclusions:**

While approximately one third of TNBC patients respond to neoadjuvant/adjuvant chemotherapy, recent studies have shown that patients with AR + TNBC are far less likely to benefit from the current standard of care chemotherapy regimens and novel targeted approaches need to be investigated. In this study, we show that activating PIK3CA mutations are enriched in AR + TNBC; and, we show that the growth and viability of AR + TNBC cell line models is significantly reduced after treatment with PI3K inhibitors used in combination with an AR antagonist. These results provide rationale for pre-selection of TNBC patients with a biomarker (AR expression) to investigate the use of AR antagonists in combination with PI3K/mTOR inhibitors.

**Electronic supplementary material:**

The online version of this article (doi:10.1186/s13058-014-0406-x) contains supplementary material, which is available to authorized users.

## Introduction

Over the past decade, the term triple-negative breast cancer (TNBC) has been used to classify tumors that lack detectable expression of the estrogen receptor (ER) and progesterone receptor (PR) and amplification of human epithelial growth factor receptor 2 (HER2). TNBC tumors are generally more aggressive than their ER + counterparts, with higher rates of relapse in the early stages and decreased overall survival in the metastatic setting [1],[2]. Although successful targeted therapies exist for ER + and HER2-amplified breast cancer, TNBC has been particularly difficult to treat given the biology of the disease has not been well understood. TNBC represents multiple independent subtypes likely requiring different therapeutic approaches, and until recently, targets for therapeutic intervention have remained elusive [3]. Current standard of care for TNBC consists of treating patients with a combination of anthracyclines and taxanes and is based on the positive results of numerous trials showing that chemotherapy combinations with these drugs, in the neo-adjuvant setting in particular, can give significant increased clinical response rates [4]. Nonetheless, there is a major need for new therapeutic options for patients suffering from TNBC.

Investigators exploring the genomic architecture of TNBCs discovered a spectrum of somatic mutations; however, only a few loci are recurrently mutated with significant frequency [5],[6]. TP53 mutations are the most frequent clonal events (62%) followed by mutations in *PIK3CA* (10.2%), the gene that encodes the p110α catalytic subunit of phosphatidylinositol-3 kinase (PI3K). Through integrated analyses of numerous world-wide gene expression (GE) datasets and a panel of TNBC lines, our laboratory provided insight into the heterogeneity of TNBC disease by identifying distinct molecular subtypes displaying unique biology that includes two basal-like (BL1 and BL2), an immunomodulatory (IM), a mesenchymal (M), a mesenchymal stem-like (MSL), and a luminal androgen receptor (LAR) subtype [3]. Of note, we demonstrated that LAR cells are in part dependent on AR signaling as siRNA-mediated AR knockdown or pharmacological inhibition of AR by bicalutamide (CDX) greatly decreases cell viability and tumor growth [3]. Also, we observed that all commercially available AR-positive (AR+) TNBC cell lines contain the PIK3CA mutation (H1047R) and are highly sensitive to the PI3K/mTOR inhibitor NVP-BEZ235 [3]. Collectively, these findings are consistent with observations that hormonally responsive cancers, such as those expressing ER [7] and AR [5],[8] are more likely to acquire PIK3CA mutations, thus prompting the experiments and discoveries reported herein.

With the goal of generating pre-clinical data that could be advanced to clinical trial design for the various subtypes of TNBC, we further investigated the molecular features of the AR + TNBC subtype, LAR. First, we discovered that PIK3CA kinase domain mutations are a frequent event in AR + TNBC clinical cases. We found that genetic or pharmacological targeting of AR in LAR cells increases the growth inhibitory activity of PI3K inhibitors. Further, we explored the combination of AR antagonism and PI3K inhibition and found an additive or synergistic effect on AR + TNBC cell growth*.* Given that AR protein expression is a reliable surrogate of LAR gene expression within TNBC, our preclinical findings provide a strong rationale for the use of AR as a biomarker for the selection of TNBC patients for clinical trials, which would investigate the efficacy of therapeutic combinations that simultaneously target AR and PI3K.

## Methods

### Acquisition of tumor tissue for this study

After the Vanderbilt University Institutional Review Board (IRB) approved the project protocol and waived the need for patient consent, de-identified TNBC tumor tissues were obtained. ER, PR and HER2 status as determined at the time of clinical evaluation was available for all specimens and was retested for the purposes of this project (see Immunostaining section below). Both frozen and formalin-fixed paraffin-embedded (FFPE) tissue samples were obtained. In addition, surgically resected tumor samples were obtained from patients with TNBC diagnosed and treated with neoadjuvant chemotherapy at the *Instituto Nacional de Enfermedades Neoplásicas* in Lima, Perú. Clinical and pathological data were retrieved from medical records under an institutionally approved protocol (INEN 10-018) and de-identified prior to distribution to Vanderbilt University Medical Center. Tumors were determined as triple-negative if they were negative for ER, PR and HER2 overexpression measured by immunohistochemistry (IHC).

### Immunostaining

Both frozen sections and FFPE tissue were used for immunohistochemical studies. For FFPE sections, antigen retrieval for both ER and PR were performed using citrate buffer (pH 6) in a decloaking chamber (Biocare Medical, Concord, CA, USA). ER (6 F11, Vector Labs, Burlingame, CA) PR (PgR636; DAKO, Carpinteria, CA) and HER2 (#2242; Cell Signaling, Danvers, MA) antibodies were utilized at 1:200, 1:50, and 1:200 dilutions, respectively. Antigen retrieval was omitted for the frozen tissues and the antibody dilutions were decreased to 1:400, 1:100 and 1:400 respectively. Visualization for both antibodies was performed using the Envision Detection System (DAKO) and 3,3-diaminobenzidine (DAB) (DAKO) as the chromogen. The percentage of invasive tumor cells with nuclear ER and PR staining and the average intensity of all positively staining tumor cells in the section were manually counted as per College of American Pathologists/American Society of Clinical Oncology (CAP/ASCO) guidelines [9]. The percentage of invasive tumor cells with membranous HER2 staining at the highest intensity level was manually assessed and recorded as per CAP/ASCO guidelines [10].

For FFPE sections, antigen retrieval for AR (DAKO, clone AR411) was performed using citrate buffer pH 6.0 in a decloaking chamber followed by 1-h incubation with antibody at room temperature at a dilution of 1:300. Antigen retrieval for p-AKT p473 (Cell Signaling) was performed using Trilogy buffer pH 6.0 (Cell Marque) in a decloaking chamber (Biocare Medical) followed by overnight incubation at 4° at a 1:25 antibody dilution. Antigen retrieval was omitted for the frozen tissues and the antibody dilutions were decreased to 1:600 and 1:30, respectively. To reduce the possibility of false negatives, tumors were determined to be AR + when >30% of the cells showed nuclear positivity of any detectable intensity level.

### Fluorescence *in situ*hybridization for HER2

Both frozen sections and FFPE tissue were used for fluorescence *in situ* hybridization (FISH). FISH for detection of possible amplification of HER2 was performed using the PathVysion HER-2 DNA Probe Kit (PathVysion Kit, Abbott Molecular, Des Planes, IL, USA) utilizing the Vysis LSI HER2/neu 17q11.2-12 SpectrumOrange™ and Vysis CEP 17 17p11.1-q11.1 SpectrumGreen Alpha Satellite DNA probes for cases with 2+ or 3+ expression by IHC. Images were visualized on a Fluorescence Olympus BX60 Microscope and analyzed using the Genus™ for Genetic Image Analysis software, version 3.6. The ratio of HER2 to CEP 17 signals was recorded and reported as an average ratio as per CAP/ASCO guidelines [10].

### PIK3CA mutation evaluation

Sanger sequencing was used to detect mutations in exons 9 and 20 of PIK3CA of PCR-amplified regions from genomic DNA. Genomic DNA was isolated from FFPE tissues of 25 AR + and 25 AR- TNBC tumors (QiaAmp FFPE, Qiagen, Valencia, CA). Genomic DNA (20 ng) was PCR-amplified in exons 9 (168 bp fragment) and 20 (159 bp fragment) using the following amplification primers: PIK3CA_ex9_F: GTTTTCCCAGTCACGACGGAAAATGACAAAGAACAGC; PIK3CA_ex9_R: CAGGAAACAGCTATGACCTGAGATCAGCCAAATTCA; PIK3CA_ex20_F: GTT TTCCCAGTCACGACGGAATGCCAGAACTACAATCTTTT and PIK3CA_ex20_R: CAGGAAACAGCTATGACTGTGTGGAAGATCCAATCCA.

### Subtyping TNBC cases and validating PIK3CA mutations in the The Cancer Genome Atlas (TCGA)

RNA-seq (lvl 3), somatic mutations (lvl3) and reverse-phase protein array (RPPA, lvl3) data for breast cancer were downloaded from TGCA (tcga-data.nci.nih.gov/). RNA-seq data (RPKM) was combined and molecularly subtyped using the online TNBCtype software according to published methods [11]. After removal of potential ER + samples, 102 TNBC samples were identified and assigned a molecular subtype, which included four ER + samples by IHC that were called ER- by RNA (Additional file 1: Table S1). Differences in RNA, protein and mutations were evaluated by Student’s *t*-test.

### Reverse-phase protein array on TNBC cell lines

Tumor or cell lysates were two-fold serial diluted for five dilutions (from undiluted to 1:16 dilution) and arrayed on a nitrocellulose-coated slide in 11 × 11 format. Samples were probed with antibodies by the Catalyzed Signal Amplification (DAKO, Carpinteria, CA) amplification approach and visualized by DAB colorimetric reaction. Slides were scanned and density quantified by MicroVigene. Relative protein levels for each sample were determined by interpolation of each dilution curve from the standard curve (supercurve) of the slide (antibody) as previously described [12]. These values were log2-transformed and normalized for protein loading by linear transformation. Linear normalized data were then median-centered for heatmap comparisons.

### Cell proliferation/viability assays and inhibitory concentration of 50% (EC50) determination

All cell lines were maintained in culture as previously described [3]. To evaluate response to dihydrotestosterone (DHT) cells were grown in charcoal-stripped (CS) media for two days prior to plating in 96-well tissue culture dishes. Media were then changed to fresh CS-media alone or increasing doses of DHT and viability determined by measuring fluorescent intensity after metabolic reduction of AlamarBlue five days later. For drug treatments, breast cancer cell lines and HMECs were seeded (3,000 to 10,000 cells) in quadruplicate wells in 96-well plates. After attachment, media was replaced with either fresh media (control) or media containing half-log serial dilutions of the following drugs: GDC-0941 (10 nM-3000 nM), GDC0980 (1 nM-300 nM), BKM120 (10 nM-3000 nM) and NVP-BEZ235 (1 nM-300 nM) purchased from Selleck Chemicals (Houston, TX) or in combination with bicalutamide (CDX) (Sigma, St. Louis, MO). Viability was determined by measuring fluorescent intensity after metabolic reduction of AlamarBlue in the presence/absence of drug incubation for 72 h. Viability assays were performed in triplicate and replicates were normalized to untreated wells. EC50 values were determined after double log-transformation of dose response curves as previously described [3].

### shRNA knockdown of AR

We transfected 293FT cells with Lipofectamine 2000 (Invitrogen, Grand Island, NY) the packaging vectors pPAX2 and pMD2.g (Addgene, Cambridge, MA) along with pLKO.1-puro Misson shRNA constructs (Sigma) targeting AR or a non-targeting control. Viral media were harvested 48 h post transfection and added to target cells along with polybrene (10 μg/mL). At 72 h post infection MDA-MB-453 (8,000 cells/well), CAL-148 (5,000 cells/well), SUM-185 (8,000 cells/well) and MFM-223 (7,000 cells/well) were seeded in quadruplicate in 96-well plates. After attachment, media were replaced with fresh media (control) or media containing half-log dilutions of (BKM120 10 to 3,000 nM, GDC-0941 10 to 3,000 nM, NVP-BEZ235 1 to 300 nM and GDC0980 3 to 1,000 nM). After incubation in drug for 72 h, viability was measured with AlamarBlue as previously described [3].

### Forced suspension viability assay

CAL-148, MFM-223, MDA-MB-453 and SUM-185 cells were plated (10,000 cells/well) in quadruplicate into 96-well plates coated with 0.9% agarose diluted in corresponding media. Following five days of growth, cells were treated with increasing half-log concentrations of GDC-0941 (10 to 3,000 nM), GDC0980 (1 nM to 300 nM), BKM120 (10 nM to 3,000 nM) and NVP-BEZ235 (1 nM to 300 nM) alone or in combination with 25 μM CDX for an additional 72 h, after which cells were imaged and viability determined by AlamarBlue.

### Immunoblotting

Cells were trypsinized, lysed and protein concentration was determined by immunoblot as previously described (12) with the following antibodies; AR polyclonal antibody, (SC-N20, Santa Cruz Biotechnology, Dallas, Texas), glyceraldehyde-3-phosphate dehydrogenase (GAPDH) (EMD Millipore, Billerica, MA) and the following antibodies from Cell Signaling pS6-Ser235/236 (#4856), S6 (#2317), p-AKT-S473 (#9271), and AKT (#9272).

### Xenograft tumor studies

Five-week-old female athymic nude Foxn1^nu^ mice (Harlan, Indianapolis, IN) were injected (subcutaneously) with either approximately 4 × 10^6^ (CAL-148) cells suspended in media (200 μL) or approximately 10 × 10^6^ (MDA-MB-453) cells suspended in 100 μL media and 100 μL matrigel into each flank using a 22-gauge needle. Once tumors reached a volume of 25 to 50 mm^3^, mice were randomly assigned to treatment or vehicle arms. Treatments included CDX per os (p.o.) (100 mg/kg/d) or GDC0980 per os (p.o.) (7.5 mg/kg/d) in 5% dimethyl sulfoxide (DMSO), 0.5% carboxymethyl cellulose (MCT) or NVP-BEZ235 per os (p.o.) (50 mg/kg/d) in 0.5% MCT as well as the combination of CDX and either GDC-0980 or NVP-BEZ235 according to the above concentrations. Tumor diameters were serially measured at the indicated times with digital calipers and tumor volumes were calculated as width^2^ × length/2. All studies were assessed for the ethical use and treatment of animals and received ethical approval by the Institutional Animal Care and Use Committee of Vanderbilt University.

## Results

### AR + TNBCs are enriched for *PIK3CA*activating mutations

**Figure 1:**
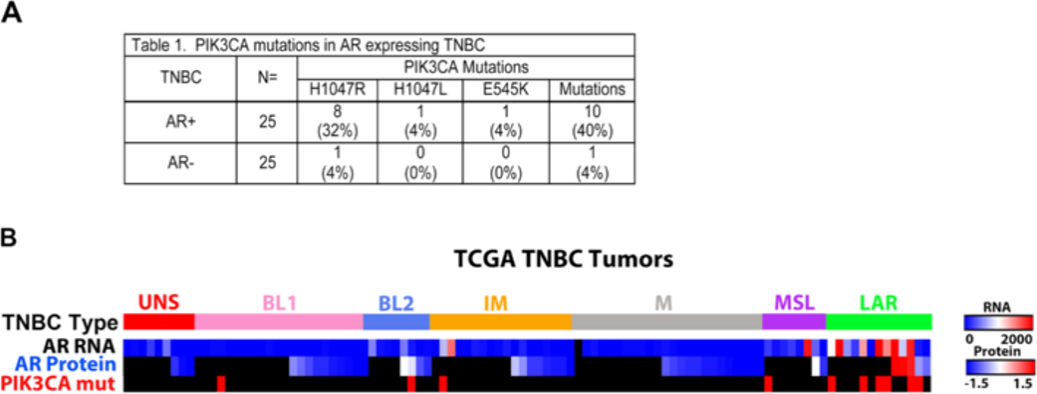
**Identification of PIK3CA mutations in androgen receptor (AR) + triple-negative breast cancer (TNBC) cell lines and tumors. (A)** Table displays the frequency of PIK3CA mutations (H1047R) in 25 TNBC AR + and 25 AR- TNBC human tumors. **(B)** Panel displays the TNBC molecular subtypes of The Cancer Genome Atlas (TCGA) breast cancer tumors with corresponding heatmaps showing the relative levels of AR RNA (RNA-seq) and AR protein (reverse-phase protein array (RPPA)). Those cases lacking protein or RNA evaluation are colored in black. The bottom row is a color bar indicating PIK3CA mutations (red) or wild-type (black) within the TCGA TNBC cases.

Previously we demonstrated high frequency of PIK3CA mutations in AR + TNBC cell lines [3]. To determine if PIK3CA mutations frequently arise in AR + TNBC, we performed Sanger sequencing on 26 AR + and 26 AR- TNBC clinical cases. We sequenced PCR-amplified regions from exons 9 and 20, the region of the gene harboring the most frequently occurring activating mutations. Consistent with our observations and ruling out *in vitro* selection for PIK3CA mutations in AR + LAR cell lines, we found that PIK3CA mutations were significantly (*P* <0.002) enriched in AR + (10/25, 40%) versus AR- TNBCs (1/25, 4%) (Figure 1A). Nearly all of the detected mutations occurred in the kinase domain (H1047R/L; 9 of 10) with only one occurring at E545K in the helical domain.

### Validation of PIK3CA mutations in AR-expressing TNBC

To verify the high frequency of PIK3CA mutations in TNBC tumors with elevated levels of AR protein, we analyzed the RNA-seq, DNA-seq and reverse-phase protein array (RPPA) data in TCGA breast cancer cohort [6]. In order to remove potential false negatives for ER, we calculated the percentile of ER gene expression for each tumor specimen and removed tumors that displayed ER expression above the 75th percentile of all genes. The resulting 102 tumors were classified according to TNBC molecular subtypes using the TNBCtype algorithm tool (Additional file 1: Table S1) [11]. The distribution of samples across the TNBC subtypes was similar to that which we had previously reported [3],[11], (UNS = 9 (8.8%), BL1 = 22 (21.6%), BL2 = 8 (7.8%), IM = 18 (17.6%), M = 24 (23.5%), MSL = 8 (7.8%) and LAR =13 (12.7%); Figure 1B and Additional file 1: Table S1). As expected, analysis of RNA-seq data generated from the LAR subtype samples confirmed significant levels of AR RNA (1,219 versus 101 reads per kilobase per million reads (RPKM), *P* <0.0001) and protein by RPPA (0.64 versus -1.15, *P* <0.0001) (Figure 1B and Additional file 1: Table S1). PIK3CA mutations were relatively infrequent (10 of 102, 9.8%) in TNBC cases overall in TCGA dataset (Figure 1B). However, when sorted by subtype, PIK3CA mutations were significantly enriched among LAR TNBCs (6 of 13, 46.2%) compared to all other TNBC subtypes (4 of 89, 4.5%, *P* <0.0001), with a predominance of H1047R mutations, consistent with our findings above (Figure 1B).

We evaluated the allele frequency of mutant and wild-type alleles in three AR + TNBCs containing H1047R PIK3CA. Since DNA-seq data were not available for two samples, we determined if allele frequencies in RNA-seq were comparable to DNA-seq from a tumor sample with both types of data (TCGA-C8-A12L). DNA-seq and RNA-seq (86% versus 77%) produced similar mutant allele frequencies, suggesting that RNA-seq could provide a reliable estimate of allele frequency (Additional file 2: Figure S1A). All three AR+/PIK3CA mutant TNBCs demonstrated a high allele frequency for the H1047R mutation (77%, 67%, and 53%), greater than the expected frequency for a heterozygous mutation (in a tumor cellularity >80%), suggesting that either the other allele had lower expression or that the mutant allele was amplified (Additional file 2: Figure S1B). We further evaluated the DNA copy number (CN) of 14 MB of DNA sequence surrounding the PIK3CA locus. CN analysis showed that the PIK3CA locus was amplified (1.544, 1.436, and 1.316, log2) in AR+/PIK3CA mutant tumors, including one tumor (TCGA-C8-A12L) with a focal amplification of only 12 genes (Additional file 3: Figure S2). The concurrent mutation and amplification of PIK3CA frequently occur in all of breast cancer with 42% (18 of 43) of tumors with amplified PIK3CA also harboring a PIK3CA mutation (Additional file 3: Figure S2).

### LAR TNBC cell lines stimulated by dihydrotestosterone have increased PI3K pathway activity and are sensitive to PI3K/TOR pathway inhibitors

**Figure 2:**
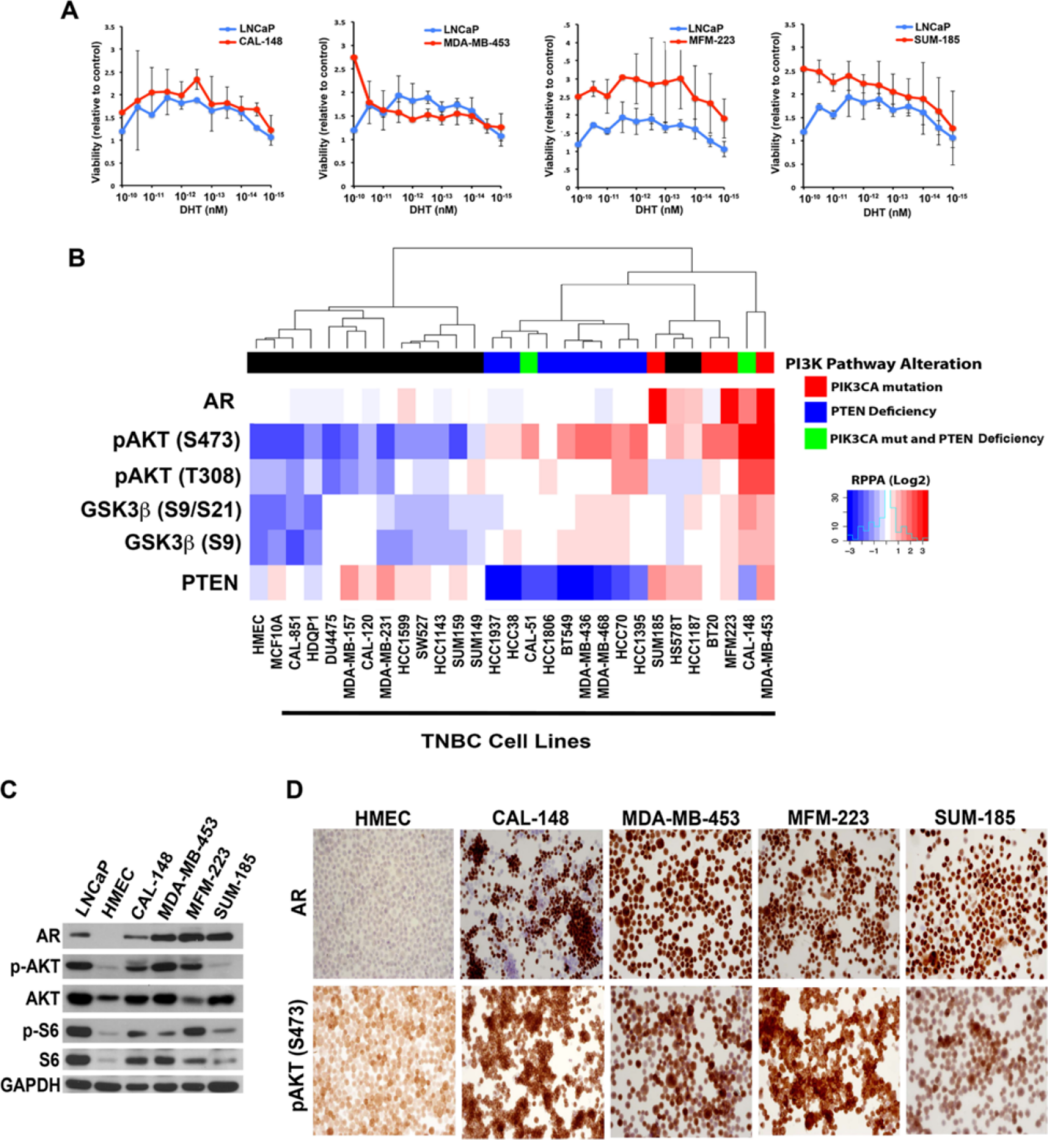
**Androgen receptor (AR) + triple-negative breast cancer (TNBC) cell lines have activated PI3K signaling and respond to dihydrotestosterone** **(DHT) stimulation (A)** **Graphs display relative viability of the prostate cancer cell line (LNCAP, red line) compared to each of the AR + TNBC cell lines (blue line) 5 days after addition of increasing doses of DHT in charcoal-stripped media.** **(B)** Heatmap displays relative protein levels (reverse-phase protein array, RPPA) of AR, p-AKT (S473 and T308), p-GSK3β (S9 and S21) and PTEN across indicated TNBC cell lines. Unsupervised hierarchical clustering was performed on PI3K pathway proteins (PTEN, p-AKT and p-GSK3β). Known PI3K pathway aberrations are indicated in the colorbar as PTEN loss (blue) or PIK3CA mutations (red). **(C)** Immunoblot displays relative protein levels of AR, p-AKT, p-S6 in AR-expressing prostate cancer (LNCaP), primary cultures of human mammary epithelial cells (HMECs) and the indicated LAR TNBC cell lines. Glyceraldehyde-3-phosphate dehydrogenase (GAPDH) serves as a loading control. **(D)** Immunohistochemistry of indicated proteins was performed on cell lines to assess levels of AR and p-AKT (S473). Results are representative of three independent experiments.

To demonstrate that AR + TNBC cell lines are responsive ligands, we measured the proliferation of the AR-dependent prostate cancer cell line LNCAP and AR + TNBC cell lines five days after treatment with increasing doses of Dihydrotestosterone (DHT). Low levels of DHT (femtomolar and picomolar concentrations) were sufficient to increase cell number two-fold in both LNCAP and AR + TNBC cell lines, demonstrating that proliferation of the cell lines can be increased by AR signaling (Figure 2A).

Since the AR + TNBC cell lines have intact AR signaling and PIK3CA mutations, we used RPPA to investigate AR, PTEN, p-AKT (S473 and T308) and p-GSK3β (S21/S9) protein levels in TNBC cell lines. We assessed PI3K pathway activity as determined by relative levels of p-AKT and p-GSK3β across the cell panel and, in particular in AR-expressing, PIK3CA mutant TNBCs (Additional file 4: Table S2). Elevated PI3K signaling correlated with PIK3CA mutation and loss of PTEN in TNBC cell lines as evidenced by high expression of activated p-AKT S473 (2.18 versus -0.23, *P* = 0.007) and p-AKT T308 (1.45 versus -0.10, *P* = 0.0032), and increased levels of p-GSK3β S9 (1.00 versus -0.26, *P* = 0.0094) and p-GSK3β S21/S9 (1.12 versus -0.19, *P* = 0.0026) (Figure 2B). Similarly, those cell lines that are null for PTEN (BT549, CAL51, HCC1395, HCC1806, HCC1937, HCC38, HCC70, MDA-MB-436 and MDA-MB-468) displayed higher levels of PI3K signaling (p-AKT S473 1.28 versus -1.28, *P* = 0.0001, p-AKT T308 0.41 versus -0.53, *P* = 0.0018, p-GSK3β S9 0.37 versus -0.76, *P* = 0.0002 and p-GSK3β S21/S9 0.26 versus -0.59, *P* = 0.0009).

To expand on the RPPA data, we performed immunoblot analyses of the PI3K/mTOR pathway in four LAR cell lines (CAL-148, MDA-MB-453, MFM-223, and SUM-185), as well as LNCaP cells (positive control) and primary cultures of human mammary epithelial cells (HMEC; negative control)) (Figure 2C). AR was expressed in the LAR TNBC cell lines at levels equal to or greater than LNCaP cells. With the exception of SUM-185 cells, AR + TNBC cell lines displayed higher levels of p-AKT (S473) and phosphorylated ribosomal S6 than human mammary epithelial cells (HMECs), suggesting activation of PI3K and downstream mTOR, respectively (Figure 2C).

To evaluate the uniformity of AR expression, we performed IHC for AR and p-AKT (S473) on LAR cell lines (Figure 2D). The AR-expressing cell lines displayed differing percentages of cells positive for AR protein expression (CAL-148: approximately 60%, MDA-MB-453: >95%, MFM-223: >95% and SUM-185: >95%). The lower percentage of AR + cells in the CAL-148 cell line is consistent with decreased levels by immunoblot analysis. Furthermore, the IHC analysis for p-AKT had similar trends to SUM-185 and HMECs, having low levels in both IHC and immunoblot analysis (Figure 2D). To determine if AR + cells displayed activated AKT, we performed dual immunofluorescence staining for AR and p-AKT (Additional file 5: Figures S3 and Additional file 6: Figure S4). The localization of p-AKT was primarily cytoplasmic, however due to the cytospin preparation and lack of adherence, localization is atypical. Regardless, nearly all AR + cells stained positive for p-AKT. Since there was heterogeneity in AR expression, we performed fluorescence-activated cell sorting (FACS) of AR in the AR + cell lines. SUM-185 cells displayed the highest levels of AR followed by MDA-MB-453, MFM-223 and CAL-148 (Additional file 7: Figure S5A). CAL-148 cells contained distinct populations expressing low (AR^low^) and high (AR^high^) levels of AR. Sanger sequencing of PIK3CA amplicons from AR^low^ and AR^high^ sorted-cell populations revealed similar frequencies to mutant alleles in each population (Additional file 7: Figure S5B), suggesting that PIK3CA mutation is clonal; whereas there is heterogeneous expression of AR within the cell line.

**Figure 3:**
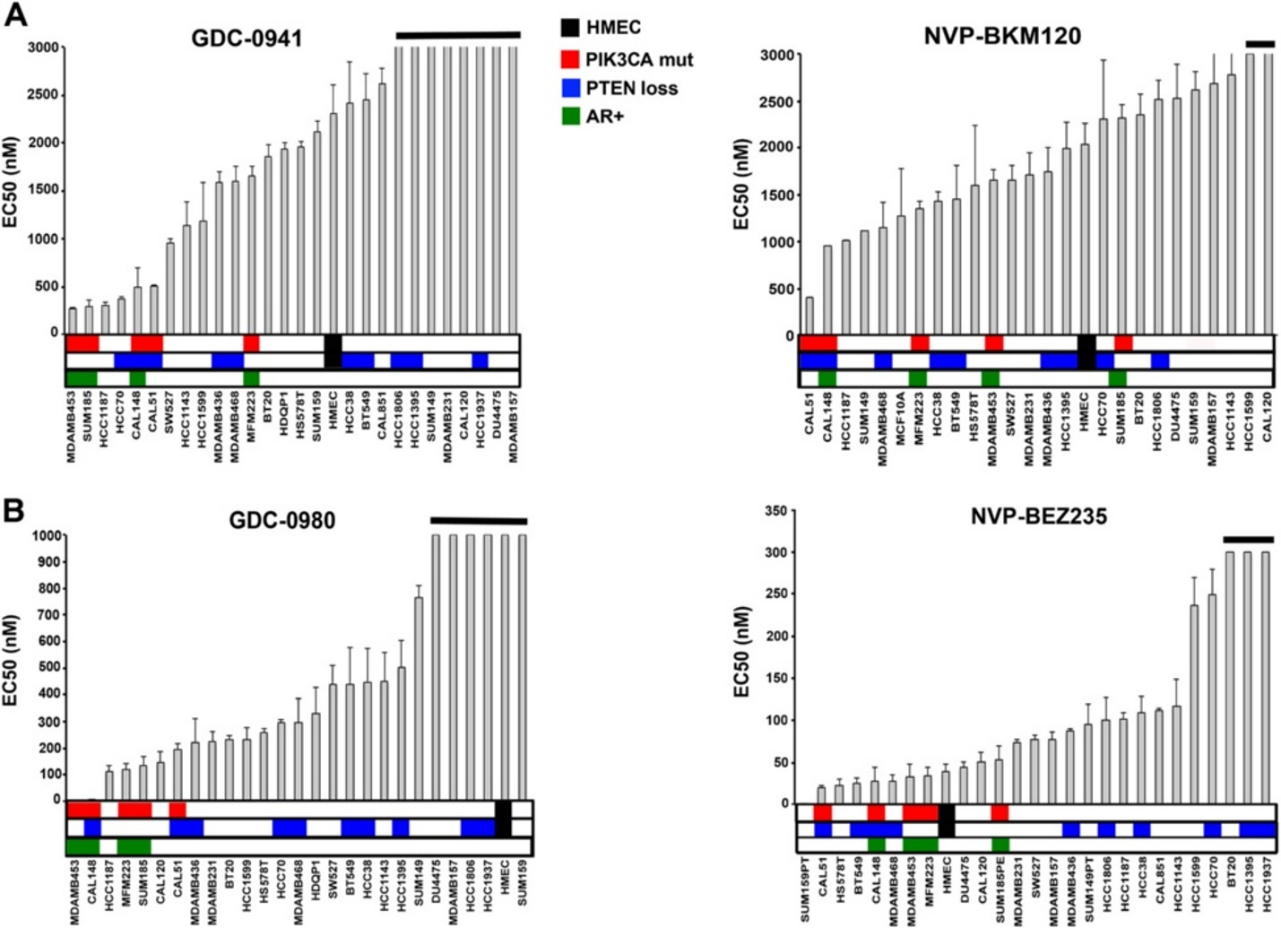
**Androgen receptor (AR) + triple-negative breast cancer (TNBC) cell lines are sensitive to PI3K inhibitors.** Bar graphs show the 50% inhibitory concentration (EC50) for TNBC cell lines treated for 72 h with single-agent **(A)** pan PI3K inhibitors (GDC-0941 and **(B)** NVP-BKM-120) or dual PI3K/mTOR inhibitors (GDC-0980 and NVP-BEZ235). Black horizontal bars above graphs indicate cell lines in which the EC50 was not reached at maximal concentration. Colorbar below indicates primary human mammary epithelial cell (HMECs) (black), AR + cell lines (green) and cell lines with PIK3CA mutations (red) or PTEN loss (blue). Error bars represent SD for three independent infections and experiments.

To determine the relative sensitivity of AR + versus AR- TNBC cell lines to PI3K pathway inhibitors, we treated a large panel of cell lines with the pan-PI3K inhibitors GDC-0941 and NVP-BKM120 or with the dual PI3K/mTOR inhibitors GDC-0980 and NVP-BEZ235. AR + cell lines (indicated in green, Figure 3) and other TNBC cell lines containing PIK3CA mutations (indicated in red, Figure 3) were the most sensitive to pharmacological PI3K inhibition, as indicated by low, half-maximal inhibitory concentration (EC50) values. AR + TNBC cells were significantly more sensitive to GDC-0941 (680 nM versus 2044 nM, *P* = 0.01) and GDC-0980 (66 nM versus 504 nM, *P* = 0.015) compared to the other cell lines. In contrast, cell lines with PTEN deficiency, while displaying elevated PI3K pathway signaling, did not uniformly have increased sensitivity to PI3K inhibitors (Figure 3). Although AR + TNBC cells displayed sensitivity to NVP-BKM120 and NVP-BEZ235, we chose to highlight the results from GDC-0941 and GDC-0980 in the figures that follow with the results from NVP-BKM120 and NVP-BEZ235 included as Additional file 8: supplemental data.

### Genetic or pharmacologic inhibition of AR increases sensitivity to PI3K pathway inhibitors

**Figure 4:**
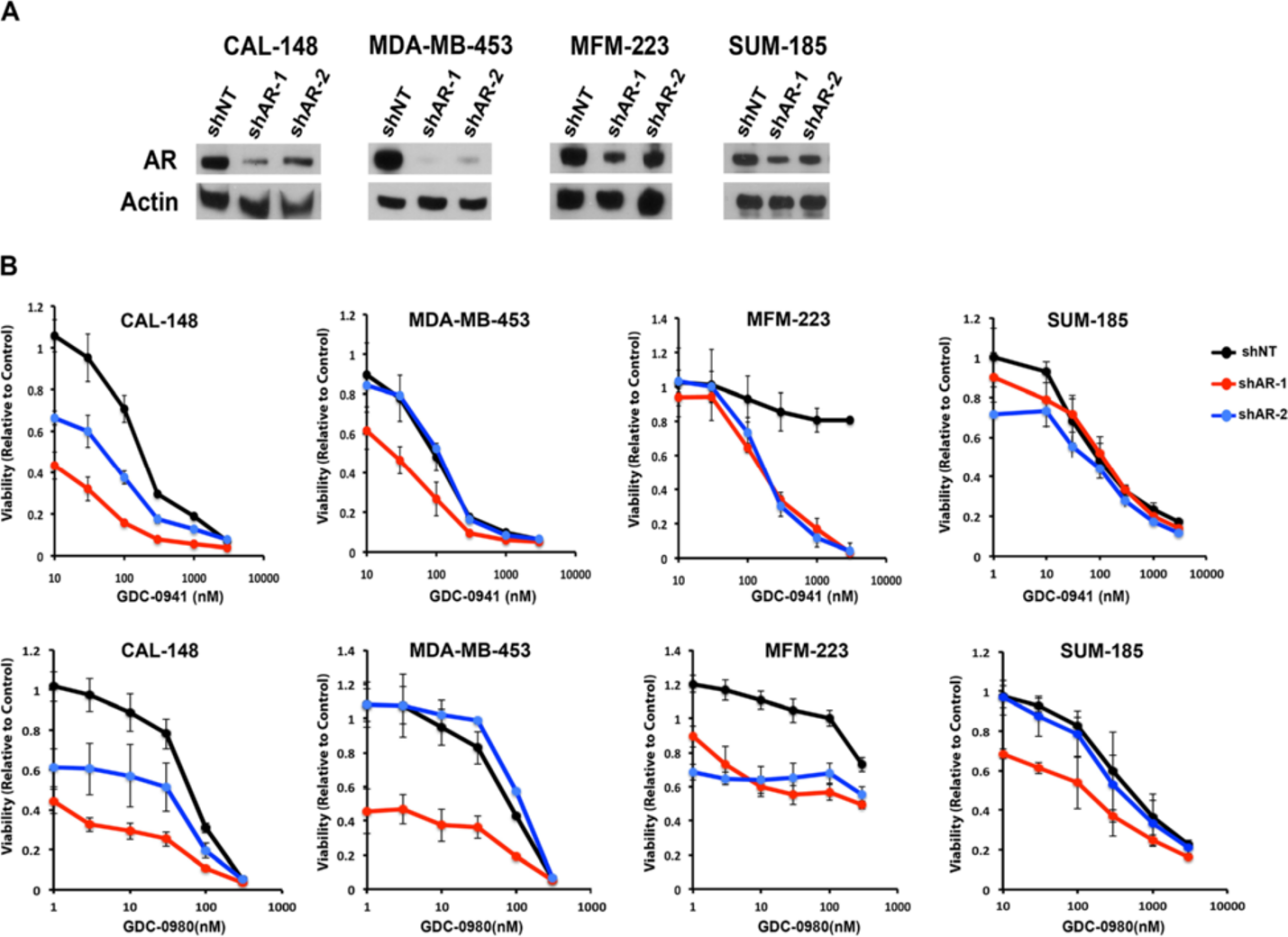
**Genetic targeting of androgen receptor (AR) is additive in combination with PI3K inhibition. (A)** Immunoblot shows AR protein levels at 72 h following transduction with two shRNAs targeting AR (shAR-1 and shAR-2) compared to transduction with nontargeting shRNA (shNT) in the indicated AR + triple-negative breast cancer (TNBC) cell lines. Actin levels are shown for the loading control. **(B)** Line graphs display relative viability of AR + cell lines transduced with nontargeting (shNT) or shRNAs targeting AR (shAR-1 and shAR-2) after 72 h treatment with increasing concentrations of the pan-PI3K inhibitor GDC-0941 (top) or the dual PI3K/mTOR inhibitor GDC-0980. Error bars represent SD for three independent infections.

Since the LAR TNBC cell lines that are sensitive to AR antagonists also harbor highly clonal PIK3CA mutations and are sensitive to PI3K inhibitors [3], we hypothesized that combined targeting of AR and PI3K would more effectively induce growth arrest or apoptosis than either agent alone. To test this hypothesis, we performed both genetic knockdown and pharmacological inhibition of AR with shRNA and CDX, respectively, in the presence and absence of PI3K pathway inhibitors. Immunoblot analysis confirmed decreased AR protein levels in all four AR-expressing TNBC cell lines infected with lentivirus containing two AR-targeting hairpins (shAR-1 and shAR-2) relative to those expressing control, non-targeting shRNA (Figure 4A). Both shRNA expression vectors targeting AR decreased cell viability alone and across increasing doses of GDC-0941 and GDC-0980 (Figure 4B). The most significant difference in viability was observed in cell lines (CAL-148 and MDA-MB-453), where the greatest knockdown of AR was achieved. Similar results were obtained with NVP-BKM-120 and NVP-BEZ235, further suggesting that the results observed were specific to inhibition of the PI3K pathway and not off-target drug effects (Additional file 9: Figure S6).

**Figure 5:**
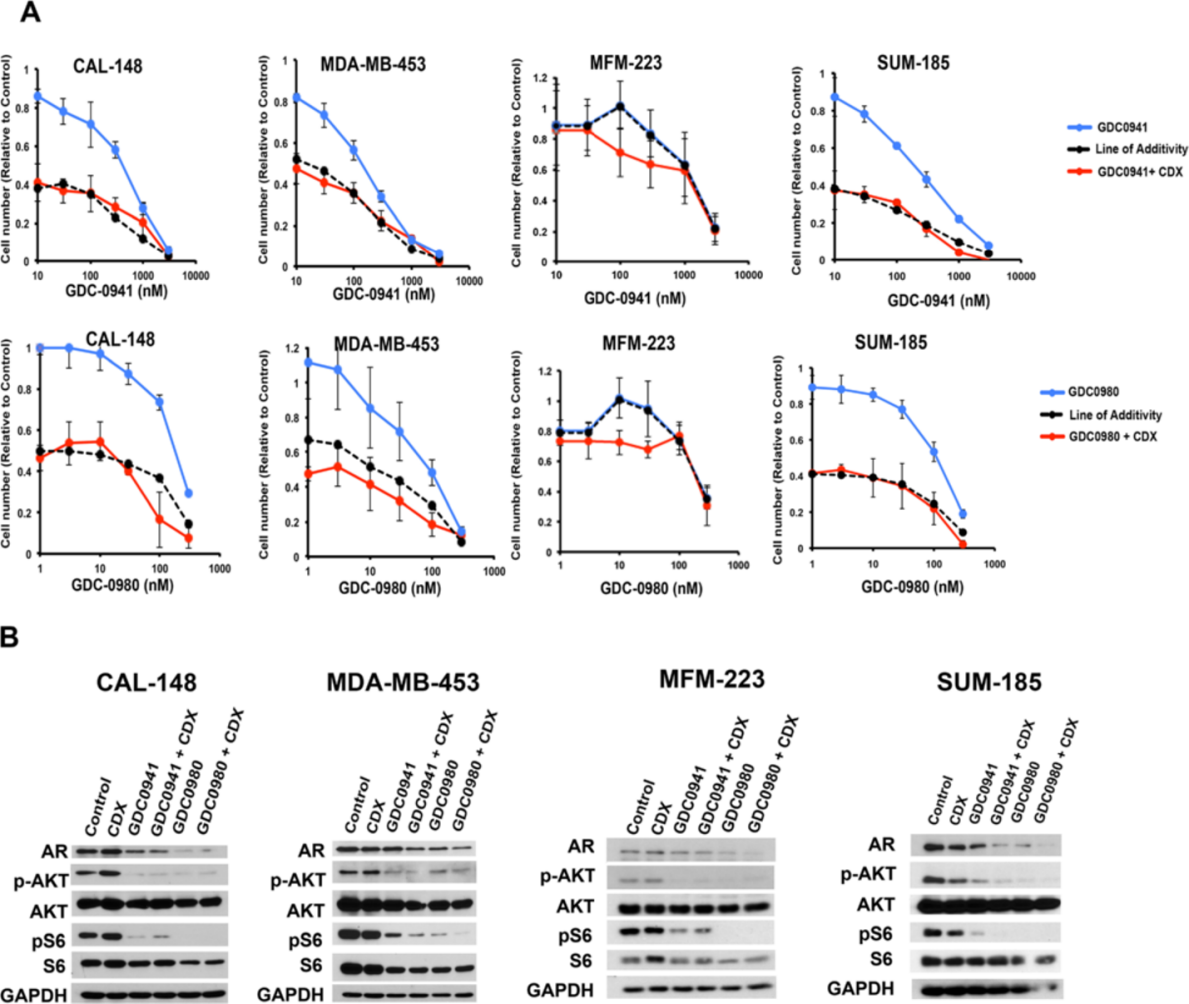
**Pharmacological targeting of androgen receptor (AR) with bicalutamide (CDX) is additive in combination with of GDC0941 and GDC0980 in AR + triple-negative breast cancer (TNBC) cell lines. (A)** Line graphs show viability of AR + cell lines treated with increasing concentrations of GDC-0941 (top) or GDC-0980 (bottom) alone (blue) or in combination (red) with 25 μM CDX. Dashed black line depicts the theoretical line of additivity of both drugs determined from the effect of CDX alone and either GDC-0941 or GDC-0980 alone. Error bars represent SD for three independent experiments. **(B)** Immunoblots from AR + TNBC cell lines treated with either CDX (25 μM), GDC-0941 (300 nM) or GDC0980 (100 nM) as single agents or CDX in combination with either GDC-0941 or GDC-0980 for 48 h analyzed for AR, p-AKT, AKT, p-S6, S6 and glyceraldehyde-3-phosphate dehydrogenase (GAPDH) protein.

Given that knockdown of AR had an additive antitumor effect with PI3K inhibitors, we pharmacologically inhibited AR using the AR antagonist CDX. Treatment with either GDC-0941 or GDC-0980 decreased proliferation/survival in a dose-dependent manner in AR + TNBC cell lines; the addition of 25 μM CDX shifted the survival curves to or below the predicted line of additivity calculated from the effect of each agent alone (Figure 5A). We observed the greatest degree of synergy with the two classes of drugs in cell lines in which CDX was less effective alone (MFM-223) as demonstrated by dose-response curves below the line of additivity (Figure 5A). In contrast, we only observed an additive inhibitory effect when the AR antagonist was used in combination with PI3K inhibitors in cell lines that were more sensitive to single agent CDX (CAL148, MDA-MB-453 and SUM185) (Figure 5A).

To verify that the PI3K inhibitors were hitting the target, we performed immunoblot analyses on lysates harvested from control cultures or cells treated alone or in combination with CDX (25 μM), GDC-0941 (300 nM) and GDC-0980 (100 nM). Treatment with GDC-0941 and GDC-0980 decreased AKT activity as assessed by levels of phosphorylated AKT (p-S473). As anticipated, GDC-0980 was more effective at decreasing mTOR activity, measured by the decrease in levels of phosphorylated S6 (Figure 5B). In contrast, CDX treatment alone had minimal effect on either the PI3K/mTOR pathway or AR levels. Interestingly, GDC-0980 treatment alone decreased AR levels and this effect could be enhanced further by the addition of CDX, suggesting that mTOR activity may contribute to elevated AR protein levels (Figure 5B), similar to the signaling observed in prostate cancer [13]. Similar results were obtained with NVP-BKM-120 and NVP-BEZ-235 (Additional file 10: Figure S7).

To determine if the observed decrease in cell number in the AR + TNBC cultures, which were treated with the combination of pathway inhibitors, was attributable to apoptotic cell death, we measured activated caspase-3 and caspase-7 levels after drug treatments (Additional file 11: Figure S8). As a positive control, we prepared lysates from cells treated with adriamycin (ADR, 3 μM) and observed caspase activation at 48 h (Additional file 11: Figure S8). While CDX treatment alone did not elevate caspase activity, treatment with single agent GDC-0941 and GDC-0980 elevated levels of cleaved caspase-3 and -7 with the levels increasing 1.2 to 2-fold when PI3K inhibitors were used in combination with CDX (Additional file 11: Figure S8A).

In addition to caspase activity, we quantified the percentage of sub 2 N DNA (sub-G1)-containing cells by FACS analysis (indicative of late stage apoptotic DNA fragmentation). In contrast to the absence of caspase cleavage describe above, CDX treatment alone led to an elevation in the sub-G_1_ fraction 48 h after treatment (Additional file 11: Figure S8B). Treatment with GDC-0941 or GDC-0980 resulted in higher sub-G_1_ fraction that increased after combination treatment with CDX (average increase 6.0% for GDC-0941 and 17.1% for GDC-0980) (Additional file 11: Figure S8B and C), concordant with the elevated caspase activity observed in Additional file 11: Figure S8A. Therefore, the decreased viability of AR + TNBC cell lines treated with a combination of AR and PI3K inhibitors can be, in part, attributed to increased apoptosis.

### Simultaneous targeting of AR and PI3K signaling decreases viability of AR-expressing cell lines grown in the absence of adhesion

**Figure 6:**
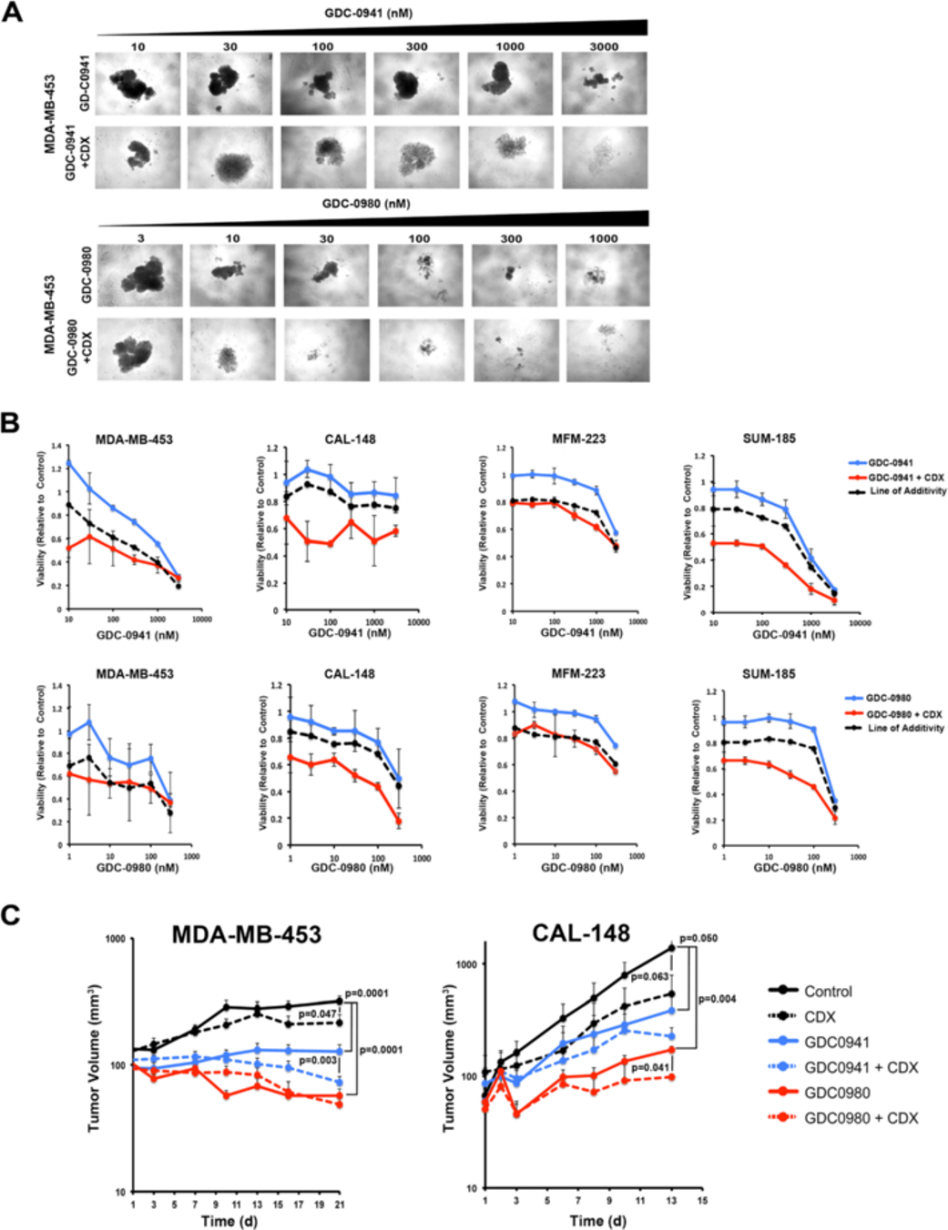
**Simultaneous pharmacological targeting of androgen receptor (AR) and PI3K decreases viability of AR + triple-negative breast cancer (TNBC) cell lines grown in 3-dimensional suspension culture or** ***in vivo*** **as xenograft tumors. (A)** Bright field images display 3-dimensional cell aggregates of MDA-MB-453 cells treated with increasing doses of GDC-0941 or GDC-0980 in the absence or presence of CDX (25 μM). **(B)** Line graphs display relative viability of 3-dimensional cell aggregates treated with GDC-0941 or GDC-0980 alone (blue) or in combination (red) with 25 μM bicalutamide (CDX). Dashed black line depicts the theoretical line of additivity determined from the effect of CDX alone and either GDC-0941 or GDC-0980 alone. **(C)** Athymic nude mice bearing established xenograft tumors from AR + TNBC cell lines (MDA-MB-453 and CAL-148) were divided into equal cohorts (n = 8) treated with either CDX (100 mg/kg/d, black hashed line), GDC-0941 (100 mg/kg/d, blue line), GDC-0980 (7.5 mg/kg/d red line) or with the combination of CDX and either GDC0-0941 (blue hashed line) or GDC-0980 (red hashed line). Serial-tumor volumes (mm^3^) were measured at the indicated days. Each data point represents mean tumor volume of 16 tumors; error bars represent standard error of the mean.

As cell viability and drug sensitivity can be different in 2-dimensional versus 3-dimensional growth conditions, we evaluated combined targeting of AR and PI3K in a forced-suspension assay devoid of matrix. Cell lines grown in suspension were treated with GDC-0941 or GDC0980 alone or in combination with CDX (Figure 6). The addition of CDX was either additive or synergistic with PI3K inhibition (Figure 6B). Similar results were obtained when PI3K was inhibited with NVP-BKM120 or NVP-BEZ235 (Additional file 12: Figure S9). However, when comparing 3-dimensional (Figure 6B) to 2-dimensional growth, we observed that viability was greater by approximately 2-fold across all doses in the 3-dimensional cultures on average. The results suggest that simultaneous targeting of AR and PI3K signaling pathways is more effective than targeting either pathway alone in AR + TNBC in monolayer or absence of adhesion in a forced suspension assay.

### Simultaneous targeting of AR and PI3K signaling decreases the growth of LAR cell line-derived xenograft tumors in athymic mice

Based on the results from 2-dimensional and 3-dimensional cell culture models, we investigated the effect of combined targeting of AR and PI3K *in vivo* in LAR cell line-derived tumor xenografts. We established MDA-MB-453 and CAL-148 tumors in athymic nude mice. When tumors reached approximately 50 mm^3^ in size, the mice were either treated with vehicle, CDX (100 mg/kg/d), GDC-0941 (50 mg/kg/d), or GDC-0980 (7.5 mg/kg/d) alone or in combinations for 13 to 21 days depending on growth rate of the cell line. Although CDX alone did not decrease tumor size, it significantly slowed growth of both MDA-MD-453 (319 versus 215 mm^3^, *P* = 0.047) and CAL-148 (1394 versus 540 mm^3^, *P* = 0.063) xenograft tumors (Figure 6C). Both single agents GDC-0941 and GDC-0980 significantly reduced tumor growth of MDA-MD-453 (319 mm^3^ versus 199 mm^3^ and 57 mm^3^ respectively, *P* = 0.0001) and CAL-148 (1394 mm^3^ versus 388 mm^3^ and 171 mm^3^ respectively, *P* = 0.050 and *P* = 0.004) (Figure 6C). Since the PI3K inhibitors were very effective alone, the added benefit of CDX was difficult to evaluate. However, the combination of CDX with GDC-0941 significantly decreased the volume of MDA-MB-453 xenograft tumors (149 mm^3^ versus 36 mm^3^, *P* = 0.003) and CDX in combination with GDC0980 significantly decreased growth (171 mm^3^ versus 97 mm^3,^*P* = 0.041) of CAL-148 xenograft tumors compared to single agent PI3K inhibition (Figure 6C). GDC-0980 alone or in combination with CDX reduced tumor growth more than GDC-0941, suggesting that concomitant targeting of mTOR provides additional therapeutic benefit or alternatively that the pharmacodynamics of the dosing protocol was superior for GDC-0980.

## Discussion

Herein, we show that activating kinase mutations in PIK3CA are a frequent event in a subset of AR + TNBC tumors and cell lines. A link between the PI3K/mTOR pathway and AR signaling was previously established in prostate cancer [14] and PIK3CA mutations are enriched in ER + breast cancer [7]. We demonstrate that not only are PIK3CA mutations predominant in AR + TNBC, but that they are co-selected with amplification of the PIK3CA locus. Concurrent amplifications and mutations in PIK3CA have previously been reported in uterine serous carcinoma [15], in uterine corpus endometriod cancer (9 of 17 amplified, 53%), head and neck squamous carcinoma (15 of 60 amplified, 25%), lung squamous carcinoma (10 of 67 amplified, 15%), and breast cancer (16 of 37 amplified, 43%) [16],[17]. The co-occurrence of amplification and mutation for PIK3CA is far more frequent than for other known oncogenes (BRAF, KRAS, ERBB2), in which mutations and amplifications tend to be mutually exclusive. This finding suggests that a higher level of PIK3CA activity may be needed to overcome a negative regulatory mechanism or that one or more genes in the 3q26 amplicon containing PIK3CA are functionally important.

Mechanistic insights to the evolution of hormone-dependent tumors with deregulated PI3K signaling were first provided by Carver *et al*., who demonstrated that inhibition of AR activates AKT signaling by reducing levels of the AKT phosphatase PHLPP in prostate cancer cells [14]. A recent siRNA screen identified AKT1 as a target that impaired the growth of AR + prostate cancer cell lines, consistent with the PI3K pathway playing an essential role in survival of AR-dependent cells [18]. Interestingly, while AR has been shown to directly inhibit PTEN transcription in prostate cancer cells [14], AR has been reported to activate PTEN transcription in breast cancer cells [19]. If the latter were the case, elevated PIK3CA protein activity (through mutation and amplification) in AR + TNBC tumors would provide a mechanism to bypass the AR-mediated upregulation of PTEN. Therefore, targeting AR alone could potentially promote survival by reducing PTEN expression and activating the PI3K pathway. Thus, our working hypothesis is that dual targeting of PI3K and AR will provide a synergistic anti-tumor effect.

We and others have shown that approximately 10 to 20% of TNBCs express AR [3],[20]. In addition, Farmer and colleagues have previously identified a distinct gene expression pattern in ER-, AR + breast cancers termed molecular apocrine representing 8 to 14% of all breast cancers [21]. Clinically, AR-negative patients have a higher likelihood of achieving pathological complete response (pCR) with neoadjuvant chemotherapy than AR + patients [22]. In addition, Masuda *et al*. recently completed a retrospective TNBC subtype analysis of gene expression performed on 130 pretreatment biopsies from TNBC patients treated with anthracycline, cytoxan and taxane. While the overall pCR response was 28%, patients with the LAR subtype had a poor response with only 10% achieving pCR prior to surgery [23]. These findings and the observation that AR + TNBC cell lines are relatively resistant to cisplatin [3], suggest TNBC patients with AR + tumors will not receive significant clinical benefit from standard chemotherapy and require novel targeted approaches.

An ongoing clinical trial evaluating CDX as a single agent in metastatic AR+, ER-/PR- breast cancers (NCT00468715/TBCRC011) has demonstrated some efficacy with a clinical benefit rate of 19% [24]. While combined inhibition of AR and the PI3K pathway has yet to be carried out in clinical trials, combinations of PI3K inhibitors with the ER downregulator, fulvestrant, are now underway in postmenopausal patients with ER+/HER2- stage IV breast cancer who have progressed on aromatase inhibitors (BELLE-2, NCT01339442) or on the TORC1 inhibitor everolimus (BELLE-3, NCT01633060) [25].

## Conclusions

The results of our study have immediate translational implications for the treatment of AR + TNBC. AR expression is already a robust biomarker with an existing CLIA-approved diagnostic platform that is used in the clinical setting. From published studies, it is apparent that patients with AR + TNBC tumors are likely to demonstrate limited benefit from the current standard-of-care chemotherapy regimens for TNBC [22],[23]. Thus, our pre-clinical data inform the design of a combination therapy that would be, to our knowledge, the first trial in which TNBC patients are divided based on a biomarker (AR expression) and, as a result, aligned to a targeted investigational combination treatment.

## Additional files

## Electronic supplementary material

Additional file 1: Table S1.: Triple-negative breast cancer (TNBC) molecular subtyping of The Cancer Genome Atlas (TCGA). Table displays TCGA patient barcode with TNBC subtype with clinical parameters (estrogen receptor (ER), progesterone receptor (PR), human epithelial growth factor receptor 2 (HER2) immunohistochemistry (IHC), HER2 fluorescent *in*-*situ* hybridization (FISH), and age) and corresponding androgen receptor (AR) protein and mRNA levels and PIK3CA mutation status. (XLSX 59 KB)

Additional file 2: Figure S1.: PIK3CA mutations have a high clonal frequency in androgen receptor (AR) + triple-negative breast cancer (TNBC) tumors. (**A**) Panels display the frequency of PIK3CA mutations (H1047R) in both DNA and RNA sequencing reads from the same tumor (The Cancer Genome Atlas (TCGA)-CE-A12L). (**B**) Panels display RNA sequencing reads from two additional AR + TNBC tumors with PIK3CA mutations. (PDF 1 MB)

Additional file 3: Figure S2.: Frequent concurrent amplification and mutation of PIK3CA in human breast cancers. Image displays 43 tumors with overlapping amplicons of PIK3CA (>4 copies) from The Cancer Genome Atlas (TCGA) breast cohort. Vertical color bars indicate androgen receptor (AR) + triple-negative breast cancer (TNBC) (green) and tumors in which PIK3CA is also mutated (blue). (PDF 869 KB)

Additional file 4: Table S2.: Linear normalized median-centered (log2) reverse-phase protein array (RPPA) values for triple-negative breast cancer (TNBC) cell lines. Table shows median-centered RPPA values for 179 proteins for each of the cell lines, performed in duplicate. (XLSX 195 KB)

Additional file 5: Figure S3.: Triple-negative breast cancer (TNBC) cell lines express both androgen receptor (AR) and p-AKT by immunofluorescence. Immunofluorescent images of MFM-223 (top) and SUM-185 (bottom) co-stained for AR (red), p-AKT(S473) (green) and counterstained with DAPI (blue). Altered cellular morphology is due to cytospin preparation. (PDF 1 MB)

Additional file 6: Figure S4.: Triple-negative breast cancer (TNBC) cell lines express both androgen receptor (AR) and p-AKT by immunofluorescence. Immunofluorescent images of MDA-MB-453 (top) and CAL-148 (bottom) co-stained for AR (red), p-AKT(S473) (green) and counterstained with DAPI (blue). Arrows indicate a percentage of AR- cells that stain positive for p-AKT. (PDF 1 MB)

Additional file 7: Figure S5.: PIK3CA mutation in CAL-148 cells is present in both androgen receptor (AR) + and AR- cells sorted by fluorescence-activated cell sorting (FACS). (**A**) FACS scattergrams for AR + triple-negative breast cancer (TNBC) cell lines incubated with alexa fluor 488 secondary antibody alone (control, top panels) or with anti-AR antibody (bottom panels). (**B**) CAL-148 cells were flow sorted into AR^low^ (bottom 20%) and AR^high^ (top 20%) populations in which DNA was isolated and PIK3CA evaluated by Sanger sequencing. (PDF 2 MB)

Additional file 8: Supplemental methods.(DOCX 101 KB)

Additional file 9: Figure S6.: shRNA targeting of androgen receptor (AR) is additive in combination with PI3K inhibitors. Line graphs display relative viability of luminal androgen receptor (LAR) cell lines transduced with nontargeting (shNT) or shRNAs targeting AR (shAR-1 and shAR-2) after 72 h treatment with the pan-PI3K inhibitor NVP-BKM-120 (top) or the dual PI3K/mTOR inhibitor NVP-BEZ235 (bottom). Data represent the average of three replicates. (PDF 865 KB)

Additional file 10: Figure S7.: Pharmacological targeting of androgen receptor (AR) with bicalutamide (CDX) is additive in combination with PI3K inhibitors in AR + triple-negative breast cancer (TNBC). (**A**) Line graphs show relative viability of AR-expressing cell lines treated with an increasing concentration of BKM120 (top) or NVP-BEZ235 (bottom) as single agents (blue) or in combination (red) with CDX (25 μM). Dashed black line depicts the theoretical line of additivity determined from the effect of CDX alone and either BKM120 or NVP-BEZ235 alone. Error bars represent SD for three independent experiments. (**B**) Immunoblots from AR-expressing TNBC cell lines treated with either CDX, BKM120 (1 μM), NVP-BEZ235 (100 nM) alone or CDX in combination with either BKM120 or NVP-BEZ235 for 24 h or 48 h analyzed for AR, p-AKT, AKT, p-S6, S6 and glyceraldehyde-3-phosphate dehydrogenase (GAPDH) protein. (PDF 2 MB)

Additional file 11: Figure S8.: Combined inhibition of androgen receptor (AR) and PI3K increases apoptotic cell death in AR + triple-negative breast cancer (TNBC) cell lines. (**A**) Bar graphs display relative caspase 3/7 activity (RLU) normalized to viable cell number 48 h after treatment with vehicle, positive control (3 μM ADR), bicalutamide (CDX) (50 μM), GDC-0941 (3 μM) or GDC-0980 (1 μM) as single agents or in combination with CDX. Error bars represent SD for three independent experiments. (**B**) Representative cell cycle histograms of the MDA-MB-453 cell line treated with similar conditions as described above. (**C**) Bar graphs indicate percentages of sub-G_1_ DNA, indicative of late-stage apoptotic DNA fragmentation. (PDF 1009 KB)

Additional file 12: Figure S9.: Simultaneous targeting of androgen receptor (AR) and PI3K decreases viability of AR + triple-negative breast cancer (TNBC) cell lines grown in a 3-D forced suspension assay. Line graphs display relative viability of 3-D cell aggregates treated with BKM120 (top) or NVP-BEZ235 (bottom) as single agents (blue) or in combination (red) with CDX (25 μM). Dashed black line depicts the theoretical line of additivity determined from the effect of bicalutamide (CDX) alone and either BKM120 or NVP-BEZ235 alone. Error bars represent SD for three independent experiments. (PDF 1 MB)

Below are the links to the authors’ original submitted files for images.

Authors’ original file for figure 1

Authors’ original file for figure 2

Authors’ original file for figure 3

Authors’ original file for figure 4

Authors’ original file for figure 5

Authors’ original file for figure 6

## Abbreviations

ADR: adriamycin
AR+: androgen receptor-positive
BL1 and BL2: basal-like
bp: base pair(s)
CAP/ASCO: College of American Pathologists/American Society of Clinical Oncology
CDX: bicalutamide
CS: charcoal-stripped
DAB: 3,3-diaminobenzidine
DHT: dihydrotestosterone
EC50: half-maximal inhibitory concentration
ER: estrogen receptor
FACS: fluorescence-activated cell sorting
FFPE: formalin-fixed paraffin-embedded
FISH: fluorescent *in*-*situ* hybridization
GAPDH: glyceraldehyde-3-phosphate dehydrogenase
GE: gene expression
HER2: human epithelial growth factor receptor 2
HMEC: human mammary epithelial cell
IM: immunomodulatory
LAR: luminal androgen receptor
M: mesenchymal
MCT: carboxymethyl cellulose
MSL: mesenchymal stem-like
pCR: pathological complete response
PI3K: phosphoinositide 3-kinase
PIK3CA: phosphatidylinositol-4,5-bisphosphate 3-kinase catalytic subunit alpha
PR: progesterone receptor
RPPA: reverse phase protein array
TCGA: The Cancer Genome Atlas
TNBC: triple-negative breast cancer
