# Supplementary figures and images for: PIK3CA mutations in androgen receptor-positive triple negative breast cancer confer sensitivity to the combination of PI3K and androgen receptor inhibitors
Source: Breast Cancer Res. 2014 Aug 8;16:406. doi: 10.1186/s13058-014-0406-x (PMC4187324; doi:10.1186/s13058-014-0406-x)

Figure S1

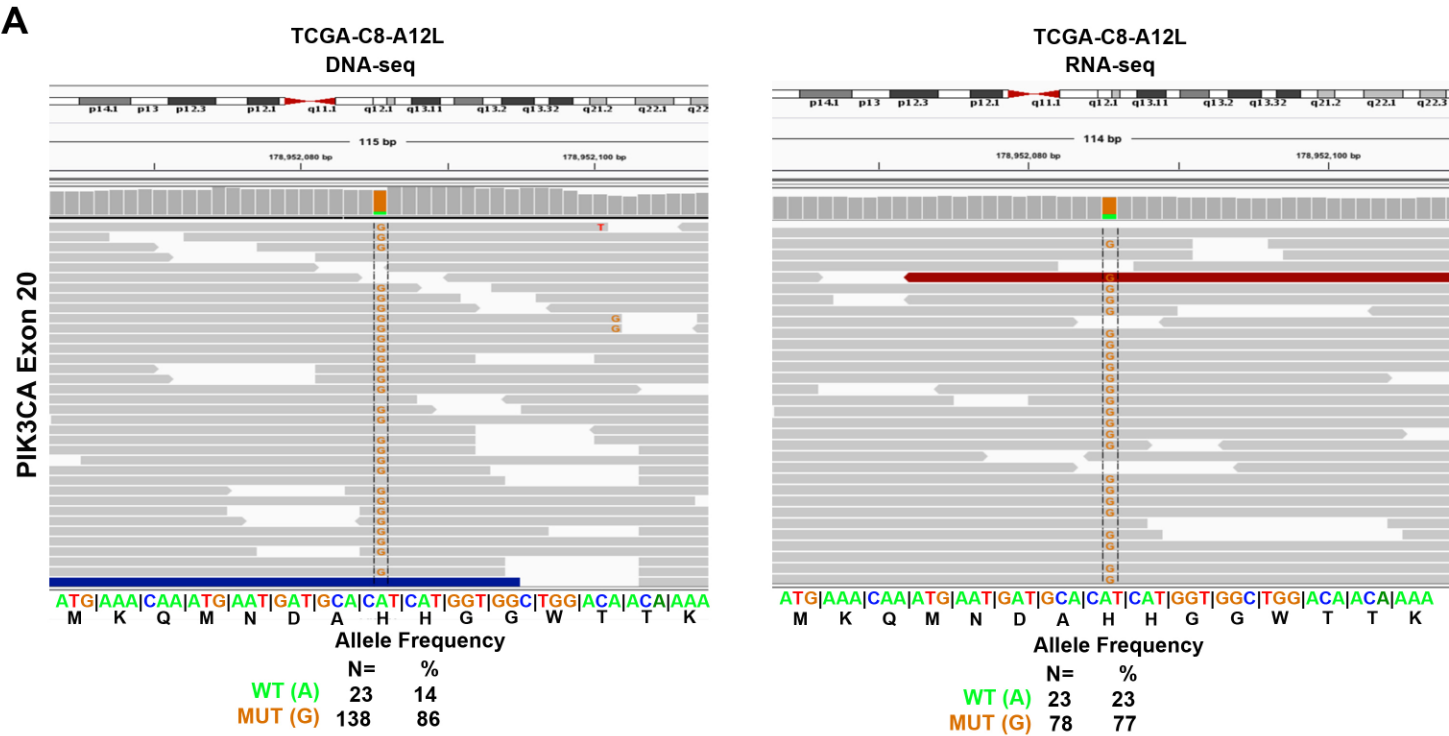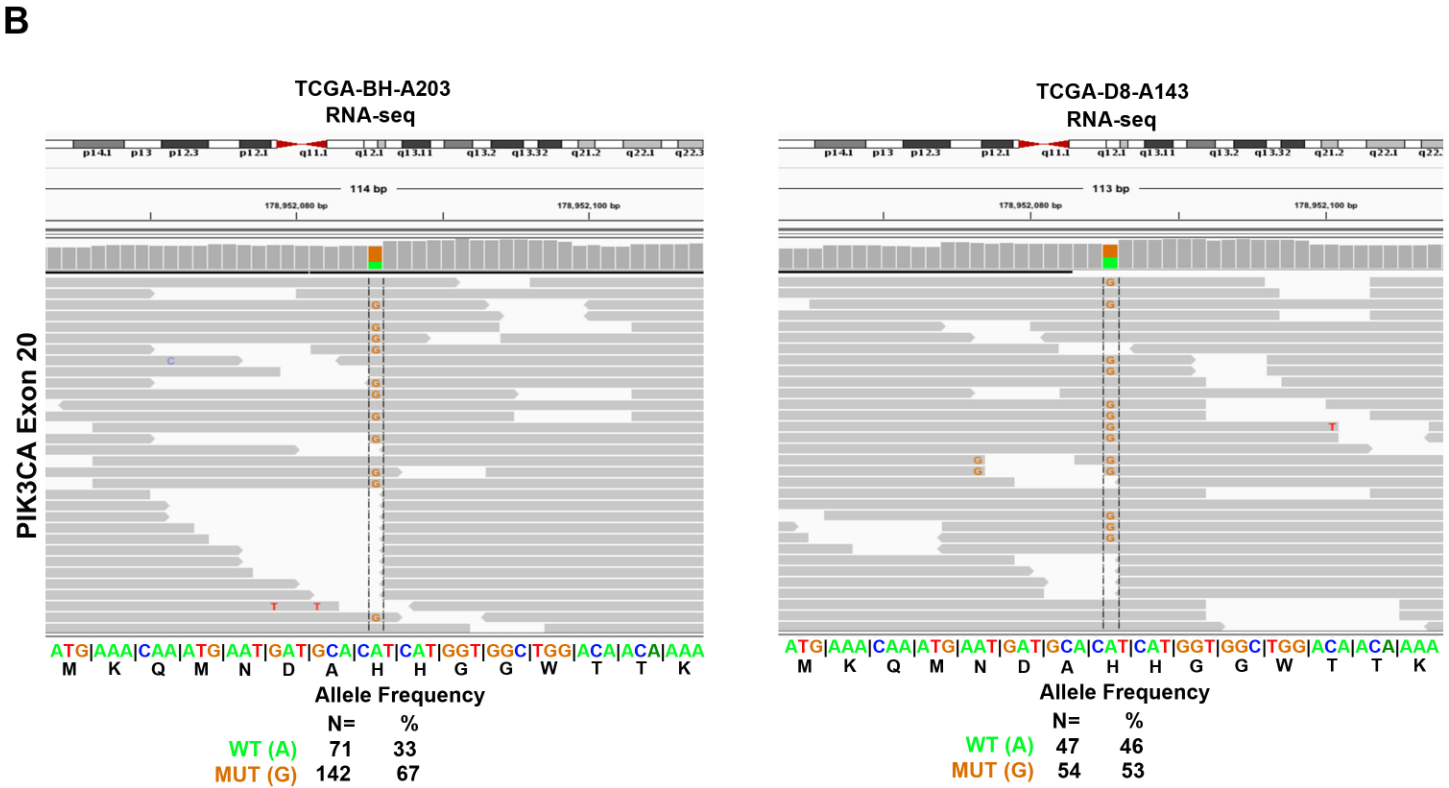

Supplement: Supplementary file 2 — Additional file 2: Figure S1.: PIK3CA mutations have a high clonal frequency in androgen receptor (AR) + triple-negative breast cancer (TNBC) tumors. (A) Panels display the frequency of PIK3CA mutations (H1047R) in both DNA and RNA sequencing reads from the same tumor (The Cancer Genome Atlas (TCGA)-CE-A12L). (B) Panels display RNA sequencing reads from two additional AR + TNBC tumors with PIK3CA mutations. (PDF 1 MB) [file 13058_2014_406_MOESM2_ESM.pdf]

Figure S2

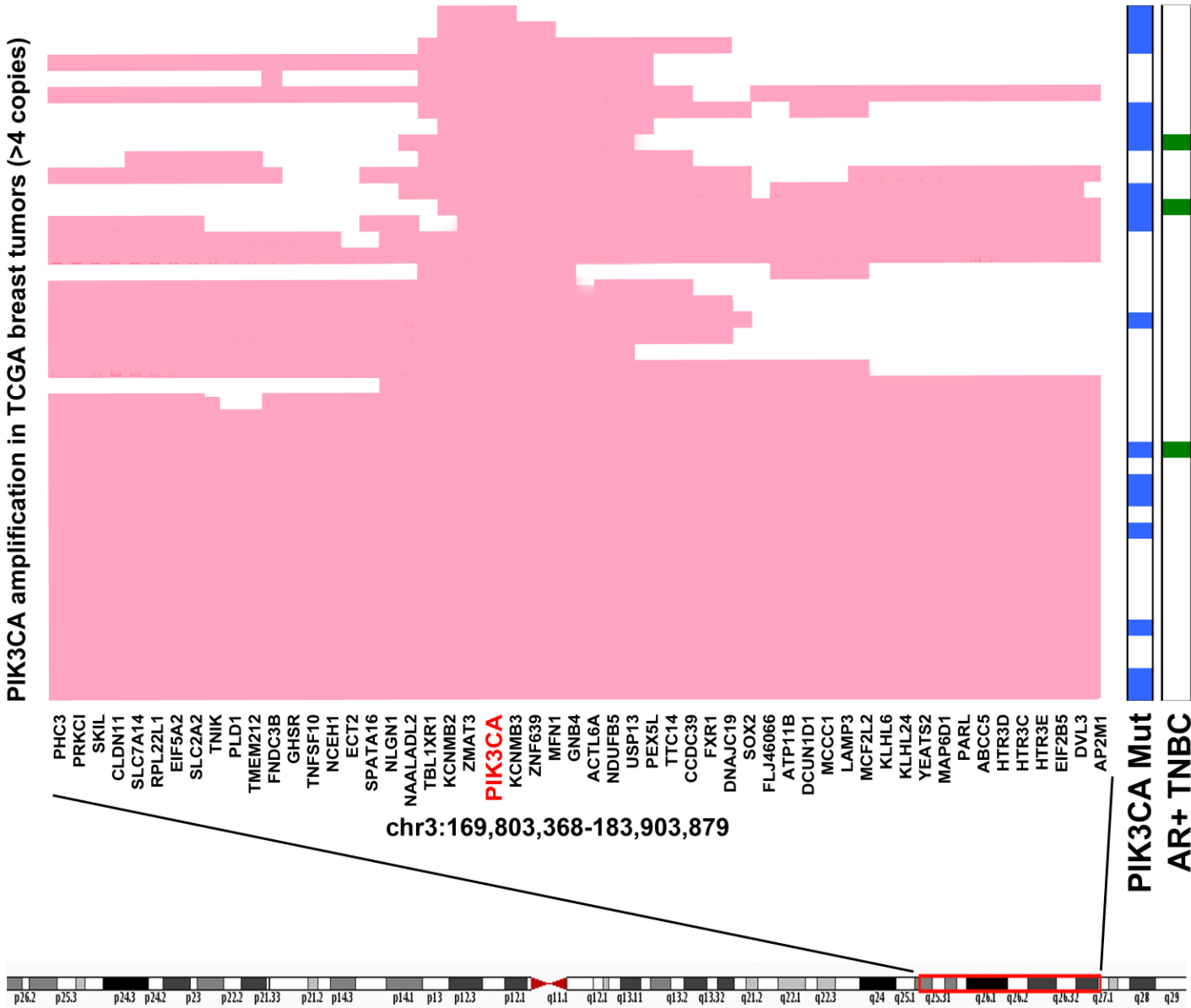

Supplement: Supplementary file 3 — Additional file 3: Figure S2.: Frequent concurrent amplification and mutation of PIK3CA in human breast cancers. Image displays 43 tumors with overlapping amplicons of PIK3CA (>4 copies) from The Cancer Genome Atlas (TCGA) breast cohort. Vertical color bars indicate androgen receptor (AR) + triple-negative breast cancer (TNBC) (green) and tumors in which PIK3CA is also mutated (blue). (PDF 869 KB) [file 13058_2014_406_MOESM3_ESM.pdf]

**Figure S3**

**MFM-223**

**AR**

**pAKT**

**DAPI**

**20X**

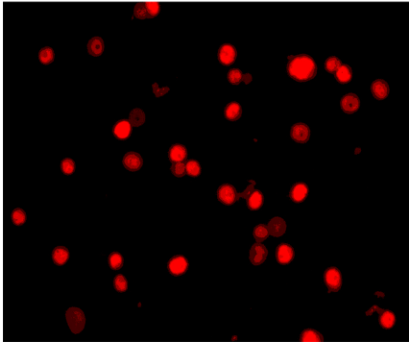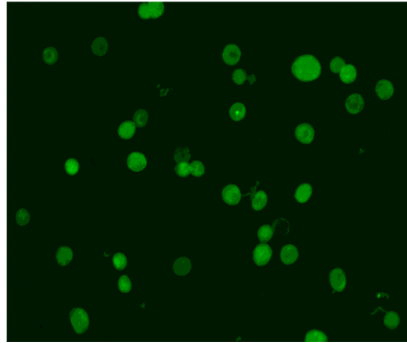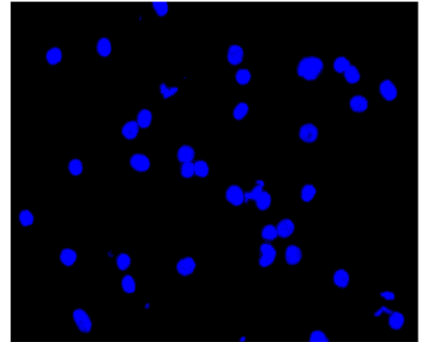

**40X**

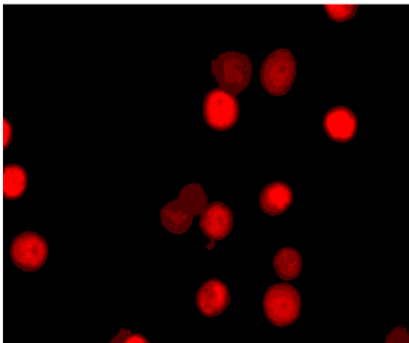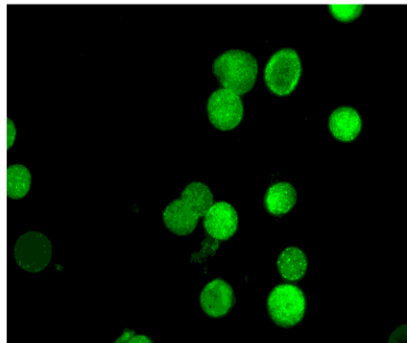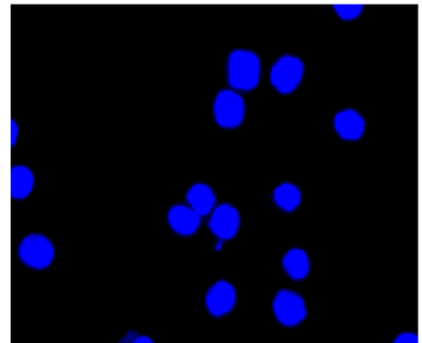

**SUM-185**

**AR**

**pAKT**

**DAPI**

**20X**

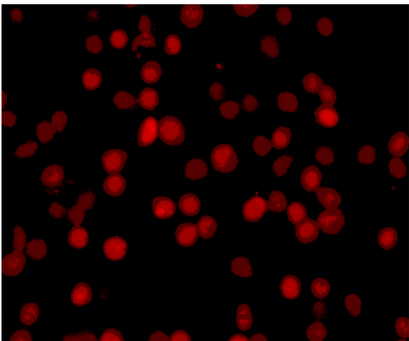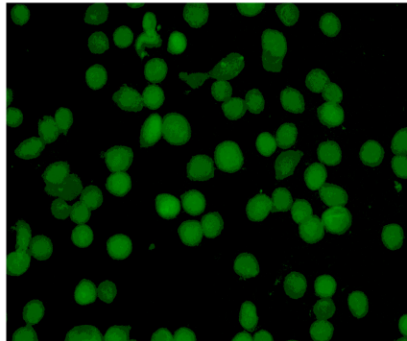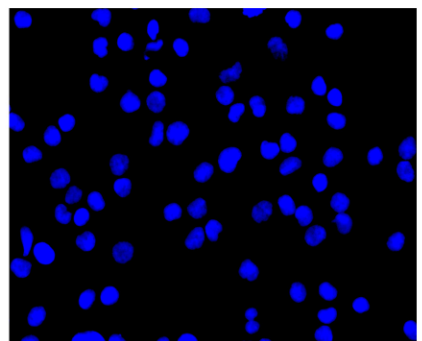

**40X**

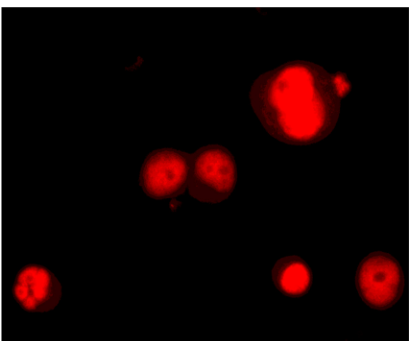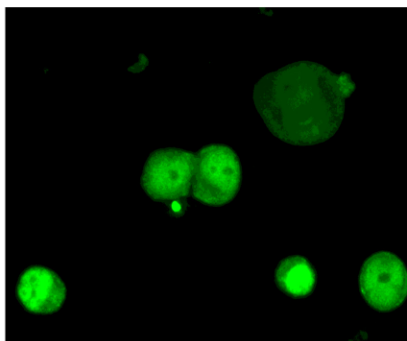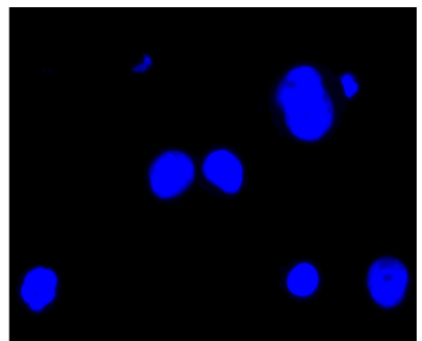

Supplement: Supplementary file 5 — Additional file 5: Figure S3.: Triple-negative breast cancer (TNBC) cell lines express both androgen receptor (AR) and p-AKT by immunofluorescence. Immunofluorescent images of MFM-223 (top) and SUM-185 (bottom) co-stained for AR (red), p-AKT(S473) (green) and counterstained with DAPI (blue). Altered cellular morphology is due to cytospin preparation. (PDF 1 MB) [file 13058_2014_406_MOESM5_ESM.pdf]

**Figure S4**

**MDA-MB-453**

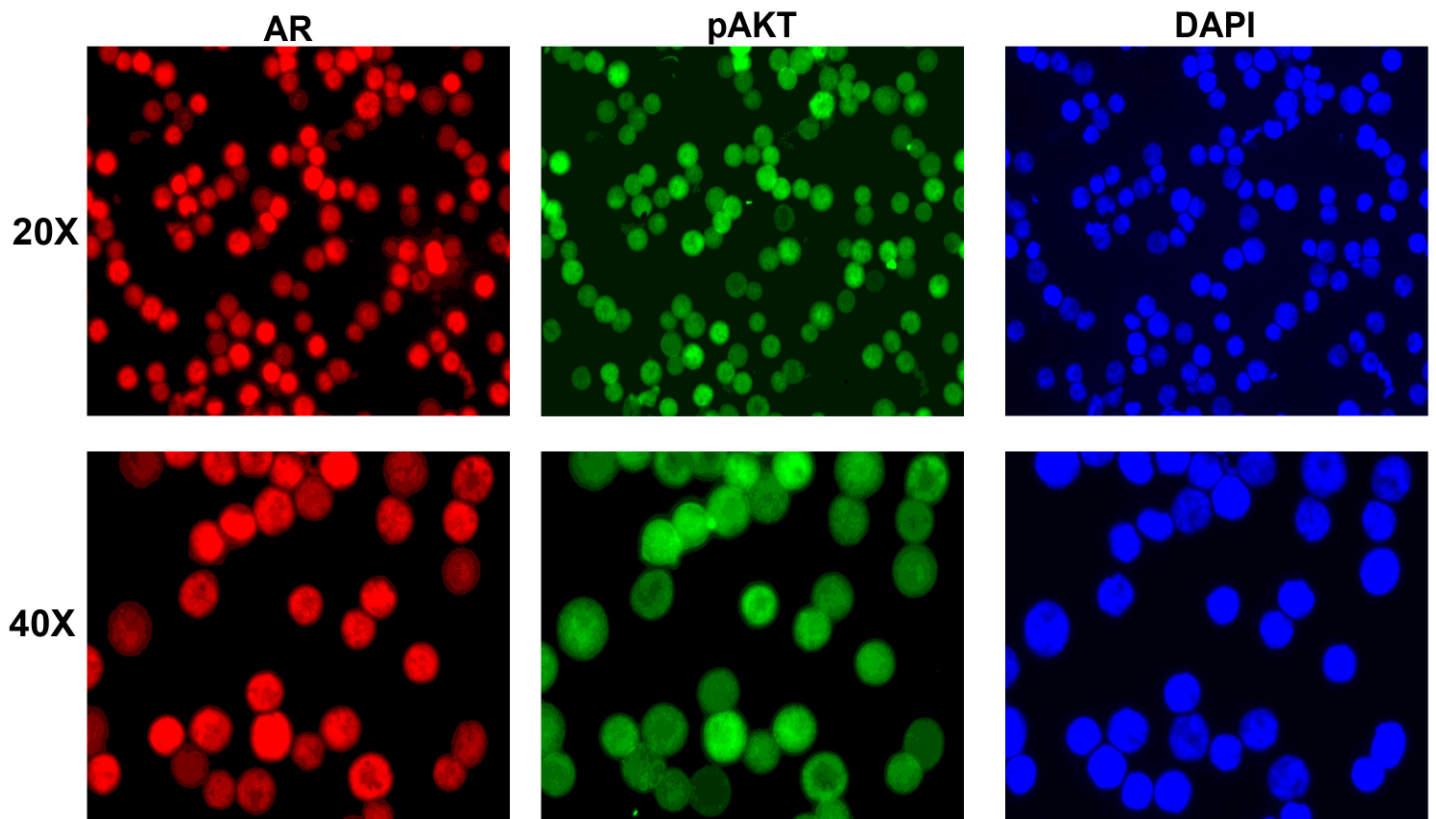

**CAL-148**

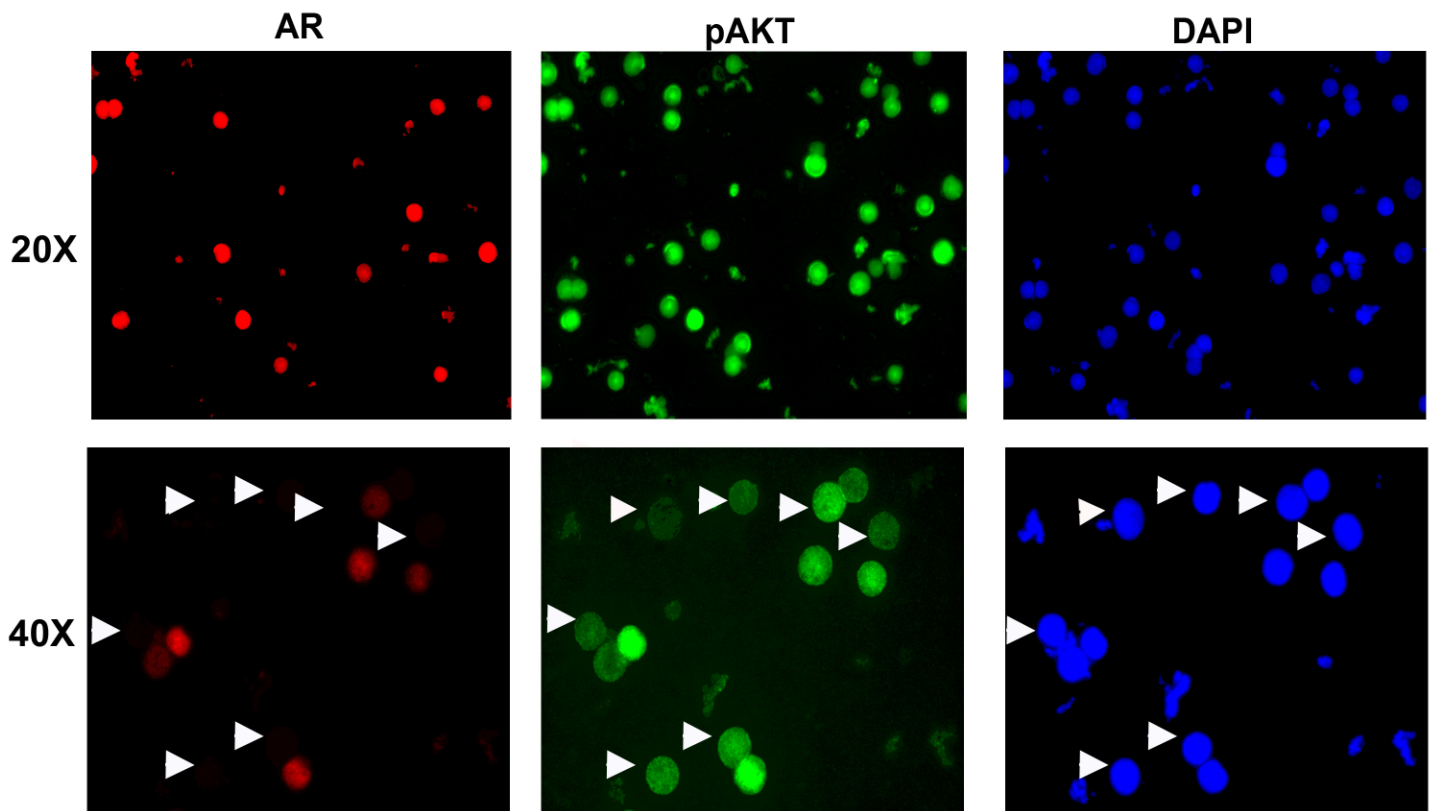

Supplement: Supplementary file 6 — Additional file 6: Figure S4.: Triple-negative breast cancer (TNBC) cell lines express both androgen receptor (AR) and p-AKT by immunofluorescence. Immunofluorescent images of MDA-MB-453 (top) and CAL-148 (bottom) co-stained for AR (red), p-AKT(S473) (green) and counterstained with DAPI (blue). Arrows indicate a percentage of AR- cells that stain positive for p-AKT. (PDF 1 MB) [file 13058_2014_406_MOESM6_ESM.pdf]

Figure S5

A

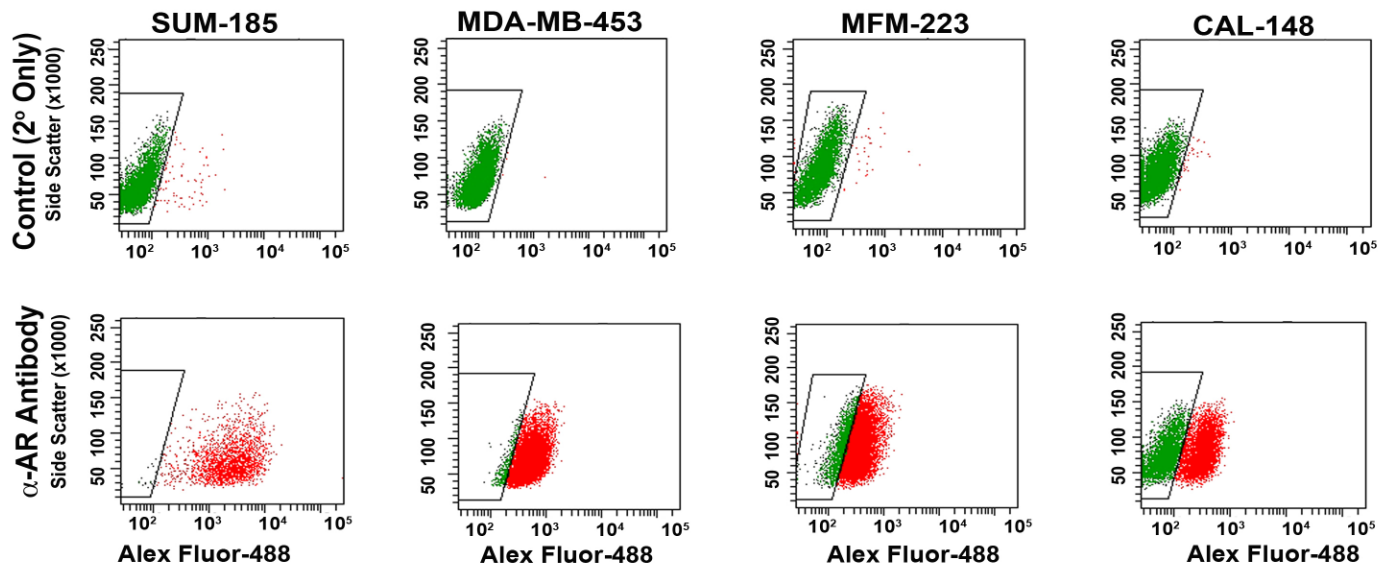

B

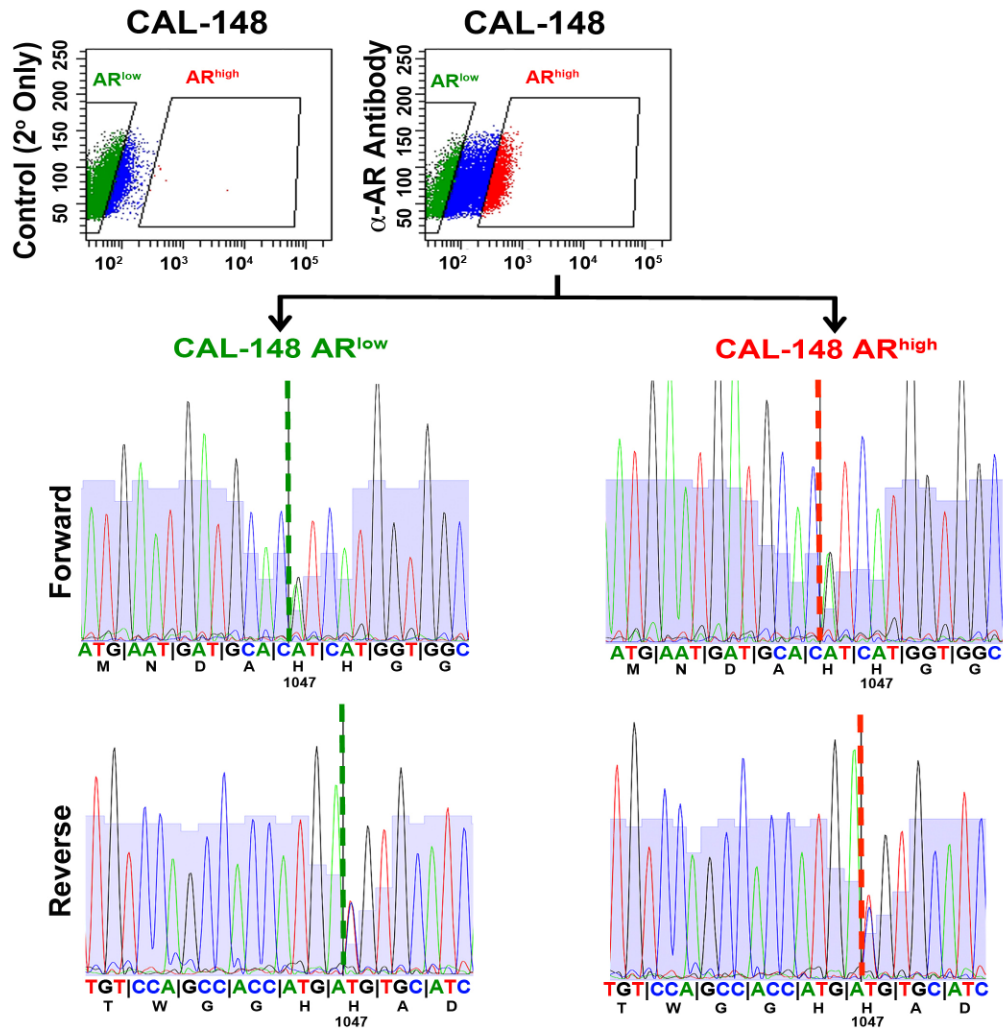

Supplement: Supplementary file 7 — Additional file 7: Figure S5.: PIK3CA mutation in CAL-148 cells is present in both androgen receptor (AR) + and AR- cells sorted by fluorescence-activated cell sorting (FACS). (A) FACS scattergrams for AR + triple-negative breast cancer (TNBC) cell lines incubated with alexa fluor 488 secondary antibody alone (control, top panels) or with anti-AR antibody (bottom panels). (B) CAL-148 cells were flow sorted into ARlow (bottom 20%) and ARhigh (top 20%) populations in which DNA was isolated and PIK3CA evaluated by Sanger sequencing. (PDF 2 MB) [file 13058_2014_406_MOESM7_ESM.pdf]

Figure S6

A

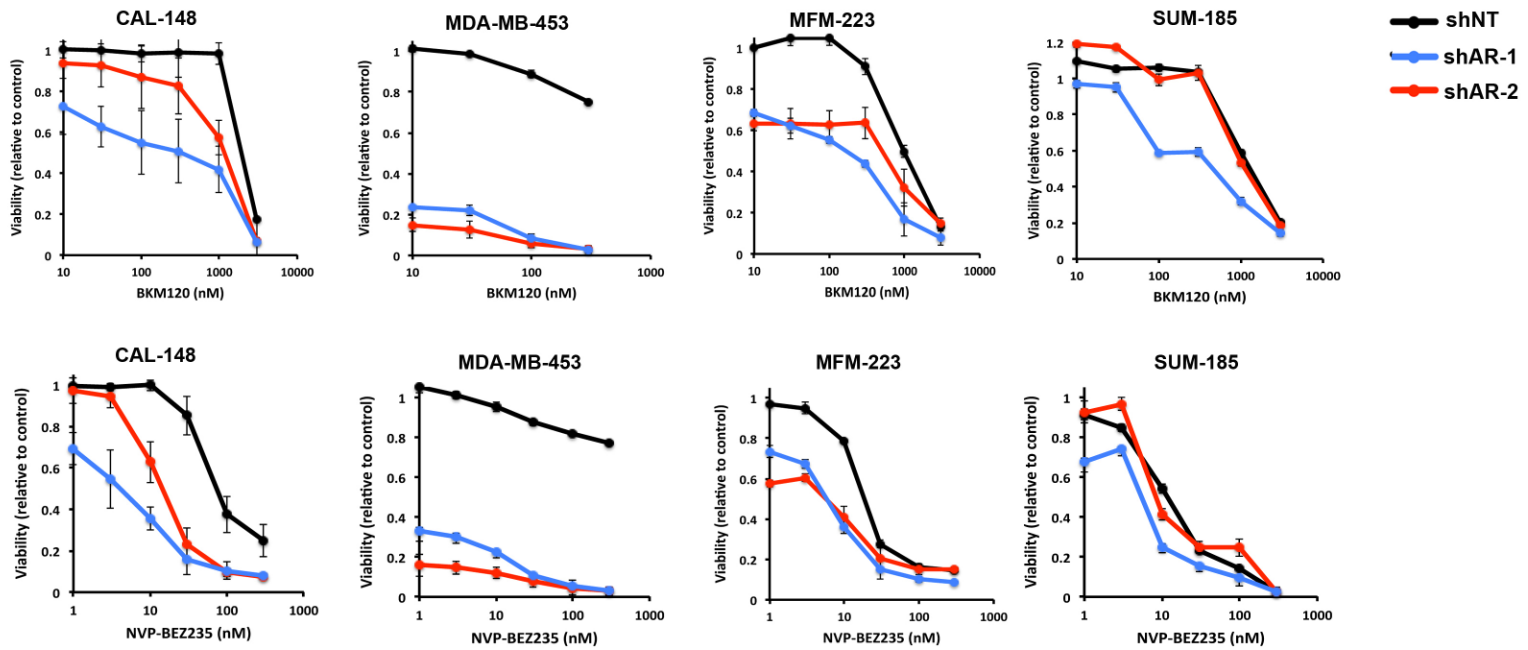

Supplement: Supplementary file 9 — Additional file 9: Figure S6.: shRNA targeting of androgen receptor (AR) is additive in combination with PI3K inhibitors. Line graphs display relative viability of luminal androgen receptor (LAR) cell lines transduced with nontargeting (shNT) or shRNAs targeting AR (shAR-1 and shAR-2) after 72 h treatment with the pan-PI3K inhibitor NVP-BKM-120 (top) or the dual PI3K/mTOR inhibitor NVP-BEZ235 (bottom). Data represent the average of three replicates. (PDF 865 KB) [file 13058_2014_406_MOESM9_ESM.pdf]

Figure S7

A

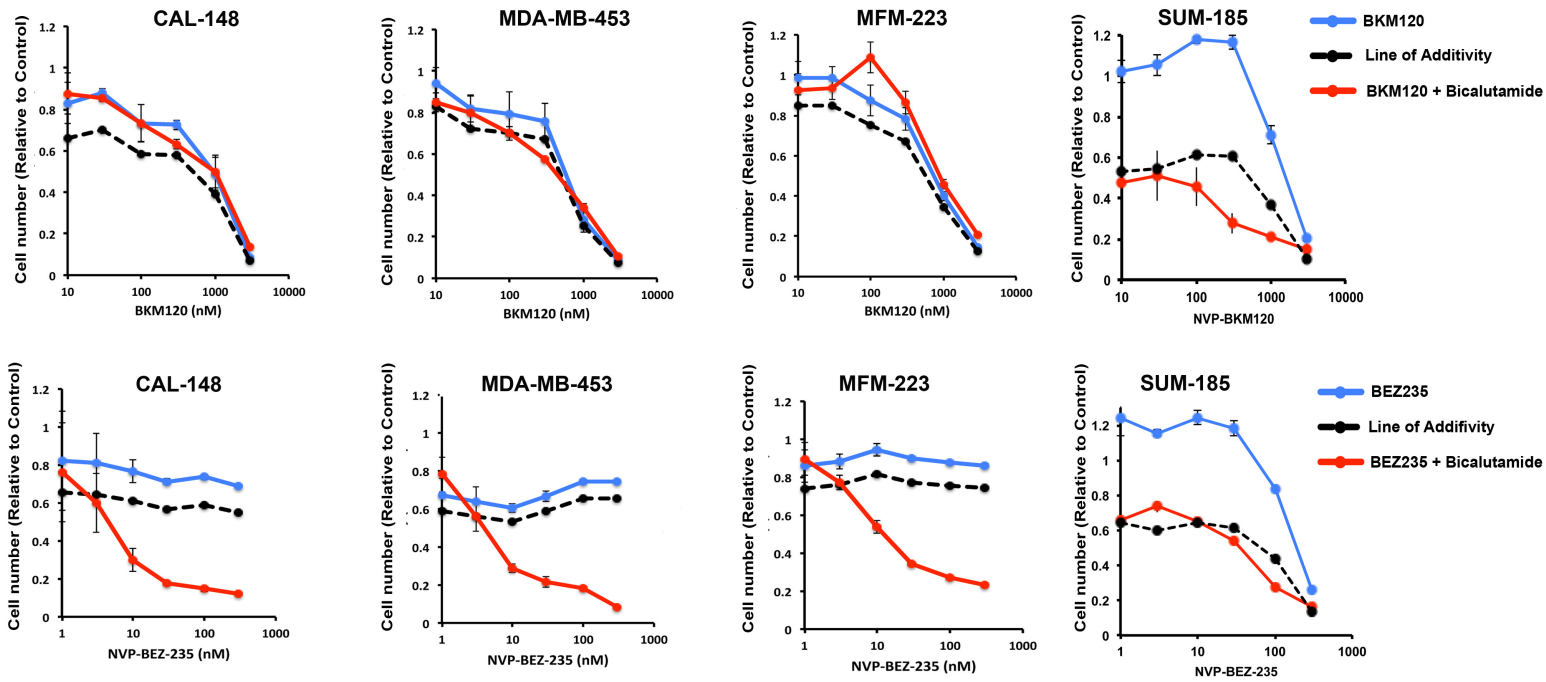

B

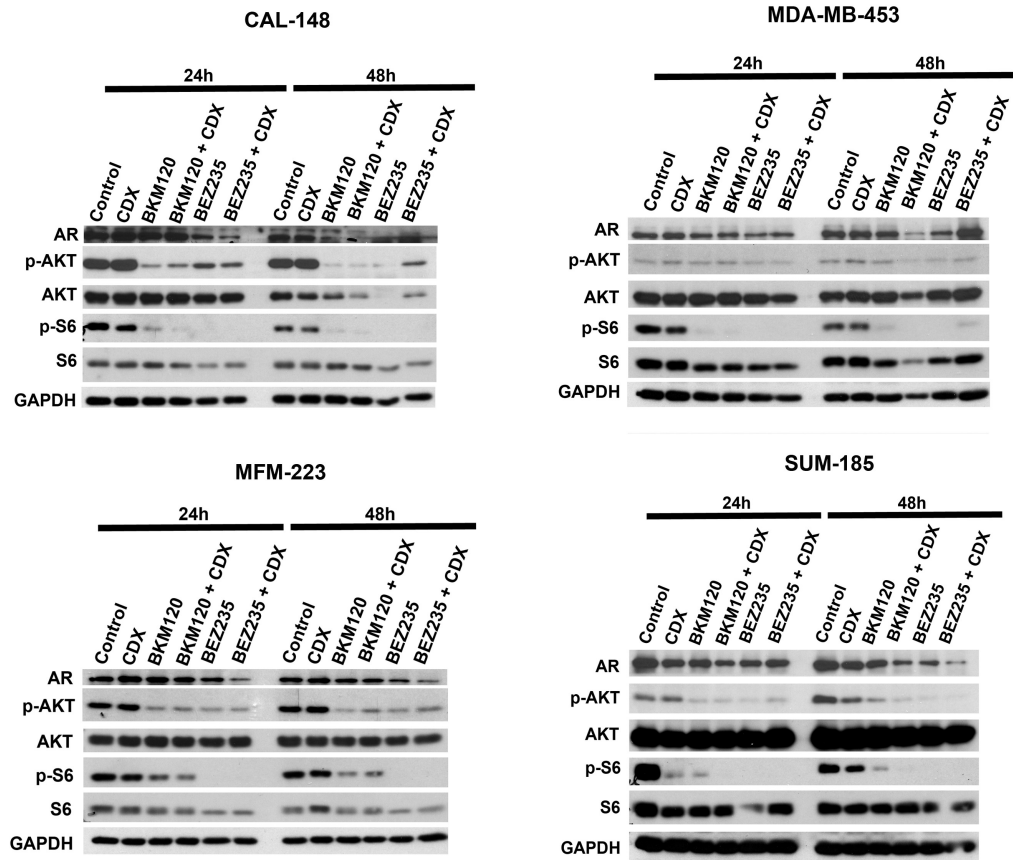

Supplement: Supplementary file 10 — Additional file 10: Figure S7.: Pharmacological targeting of androgen receptor (AR) with bicalutamide (CDX) is additive in combination with PI3K inhibitors in AR + triple-negative breast cancer (TNBC). (A) Line graphs show relative viability of AR-expressing cell lines treated with an increasing concentration of BKM120 (top) or NVP-BEZ235 (bottom) as single agents (blue) or in combination (red) with CDX (25 μM). Dashed black line depicts the theoretical line of additivity determined from the effect of CDX alone and either BKM120 or NVP-BEZ235 alone. Error bars represent SD for three independent experiments. (B) Immunoblots from AR-expressing TNBC cell lines treated with either CDX, BKM120 (1 μM), NVP-BEZ235 (100 nM) alone or CDX in combination with either BKM120 or NVP-BEZ235 for 24 h or 48 h analyzed for AR, p-AKT, AKT, p-S6, S6 and glyceraldehyde-3-phosphate dehydrogenase (GAPDH) protein. (PDF 2 MB) [file 13058_2014_406_MOESM10_ESM.pdf]

Figure S8

A

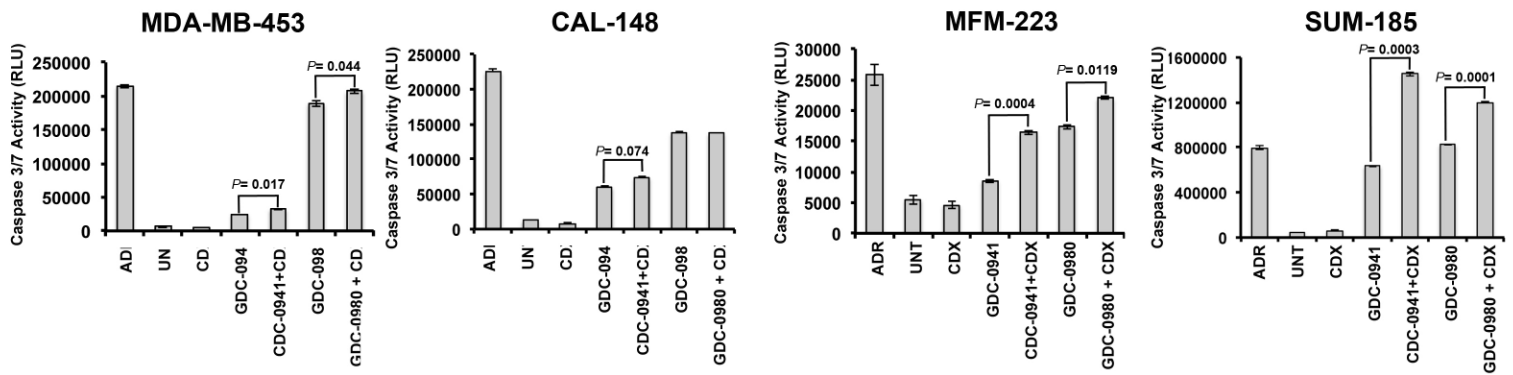

B

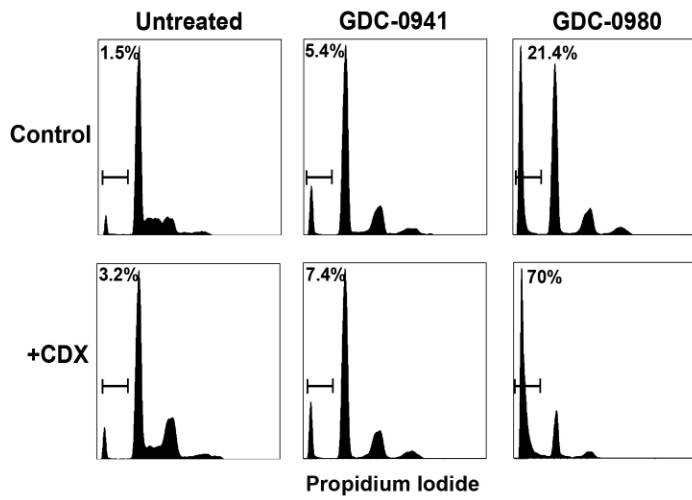

C

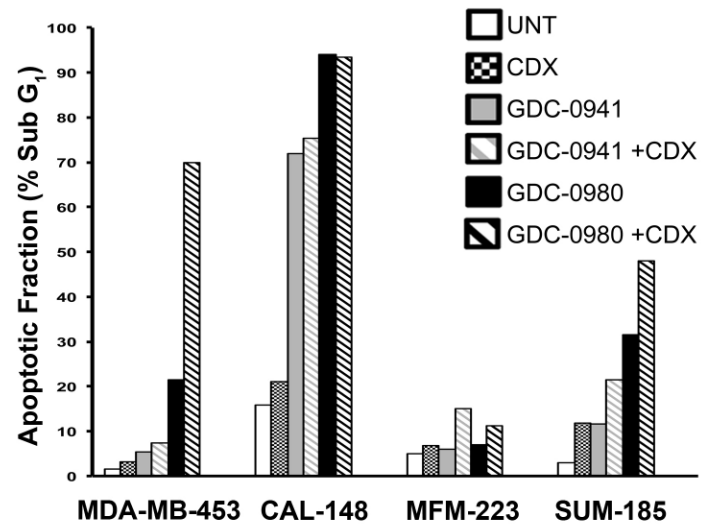

Supplement: Supplementary file 11 — Additional file 11: Figure S8.: Combined inhibition of androgen receptor (AR) and PI3K increases apoptotic cell death in AR + triple-negative breast cancer (TNBC) cell lines. (A) Bar graphs display relative caspase 3/7 activity (RLU) normalized to viable cell number 48 h after treatment with vehicle, positive control (3 μM ADR), bicalutamide (CDX) (50 μM), GDC-0941 (3 μM) or GDC-0980 (1 μM) as single agents or in combination with CDX. Error bars represent SD for three independent experiments. (B) Representative cell cycle histograms of the MDA-MB-453 cell line treated with similar conditions as described above. (C) Bar graphs indicate percentages of sub-G1 DNA, indicative of late-stage apoptotic DNA fragmentation. (PDF 1009 KB) [file 13058_2014_406_MOESM11_ESM.pdf]

Figure S9

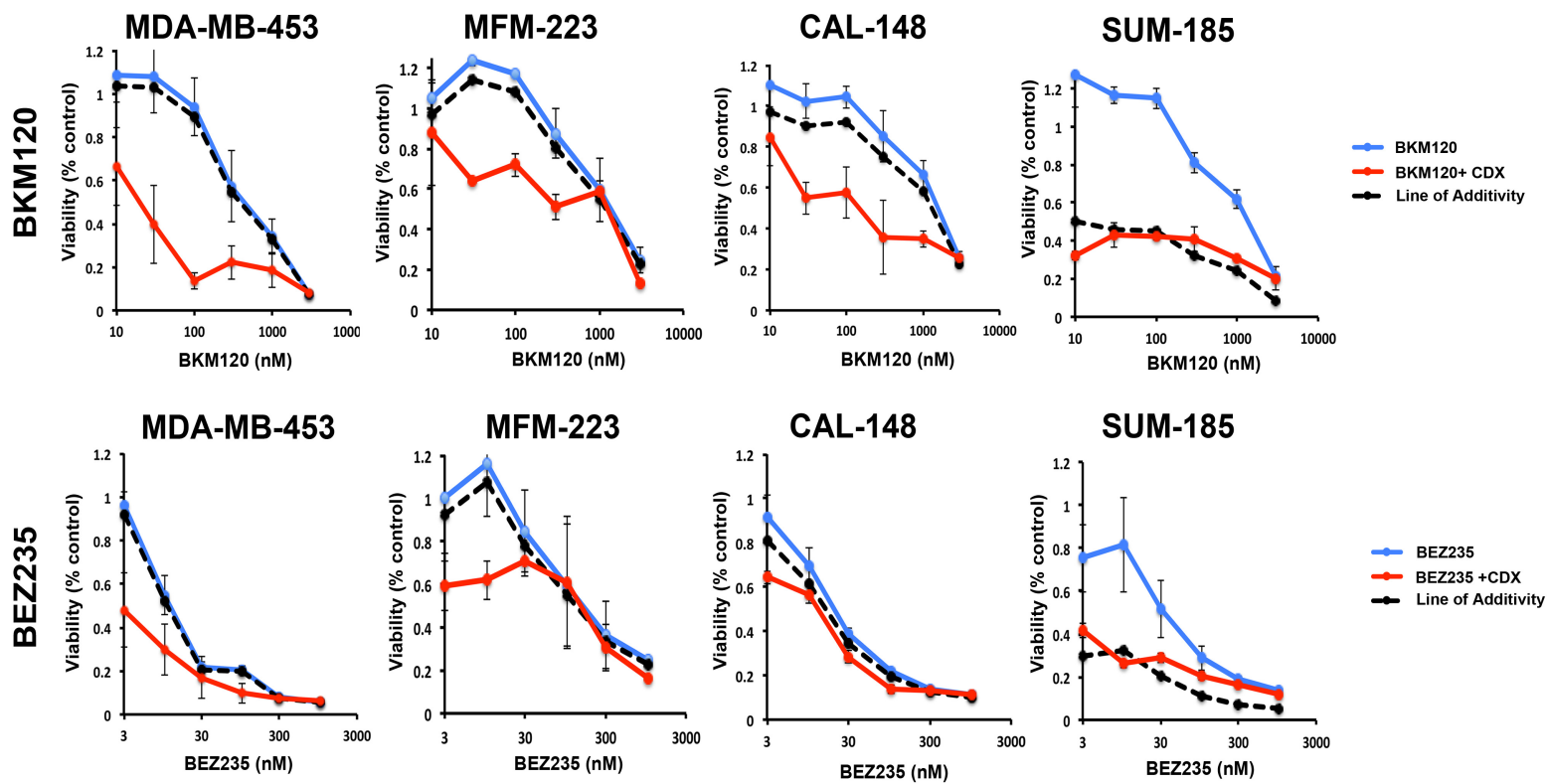

Supplement: Supplementary file 12 — Additional file 12: Figure S9.: Simultaneous targeting of androgen receptor (AR) and PI3K decreases viability of AR + triple-negative breast cancer (TNBC) cell lines grown in a 3-D forced suspension assay. Line graphs display relative viability of 3-D cell aggregates treated with BKM120 (top) or NVP-BEZ235 (bottom) as single agents (blue) or in combination (red) with CDX (25 μM). Dashed black line depicts the theoretical line of additivity determined from the effect of bicalutamide (CDX) alone and either BKM120 or NVP-BEZ235 alone. Error bars represent SD for three independent experiments. (PDF 1 MB) [file 13058_2014_406_MOESM12_ESM.pdf]

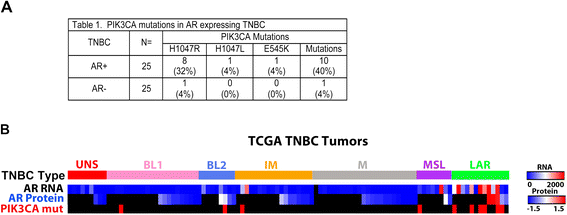

Supplement: Supplementary file 13 — Authors’ original file for figure 1 [file 13058_2014_406_MOESM13_ESM.gif]

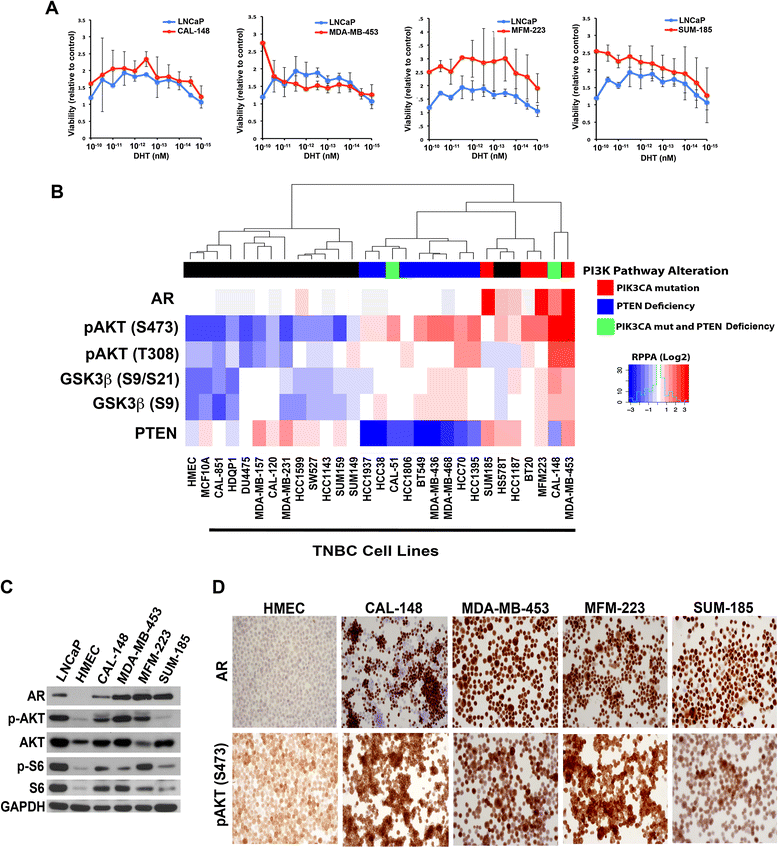

Supplement: Supplementary file 14 — Authors’ original file for figure 2 [file 13058_2014_406_MOESM14_ESM.gif]

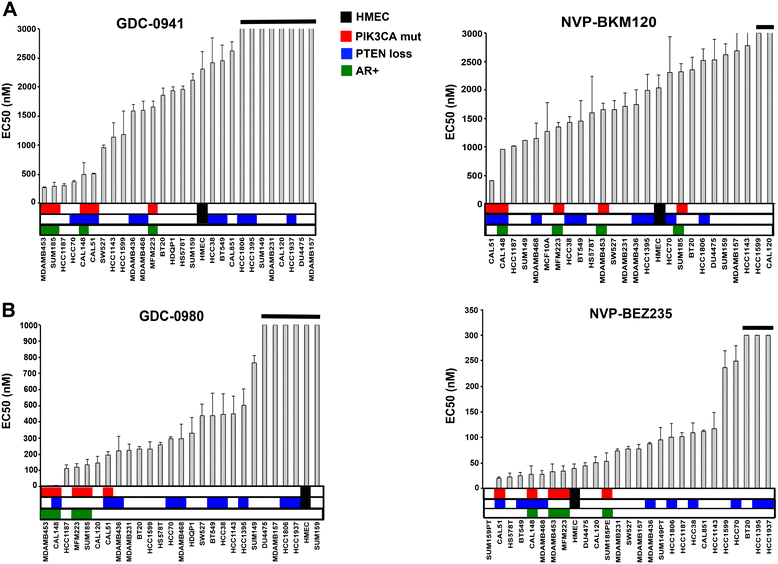

Supplement: Supplementary file 15 — Authors’ original file for figure 3 [file 13058_2014_406_MOESM15_ESM.gif]

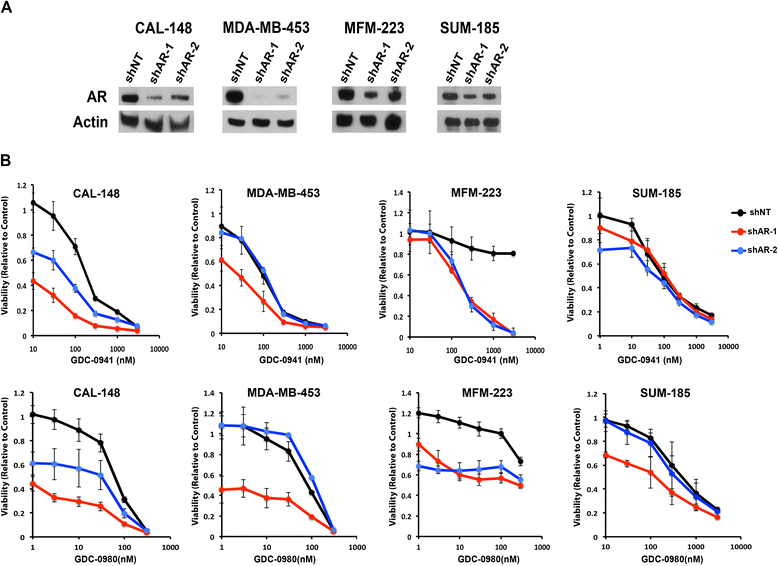

Supplement: Supplementary file 16 — Authors’ original file for figure 4 [file 13058_2014_406_MOESM16_ESM.gif]

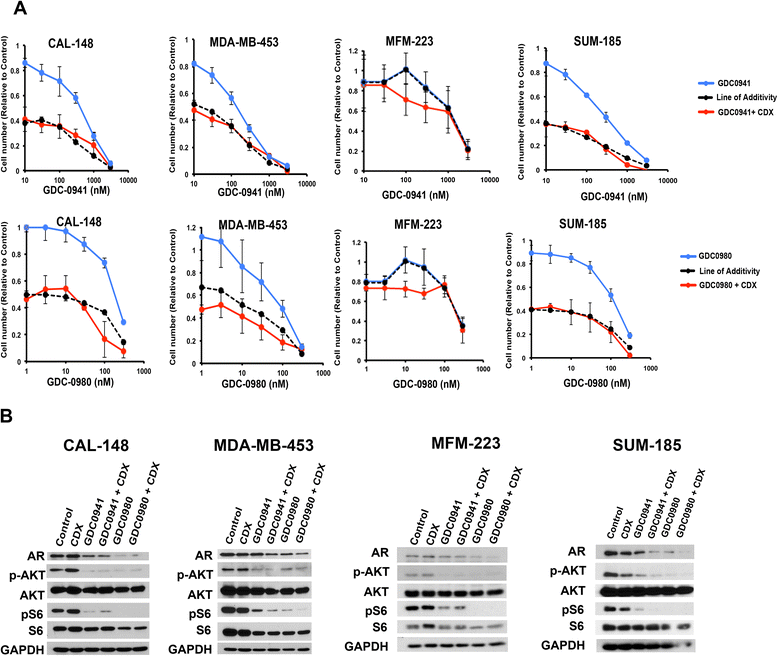

Supplement: Supplementary file 17 — Authors’ original file for figure 5 [file 13058_2014_406_MOESM17_ESM.gif]

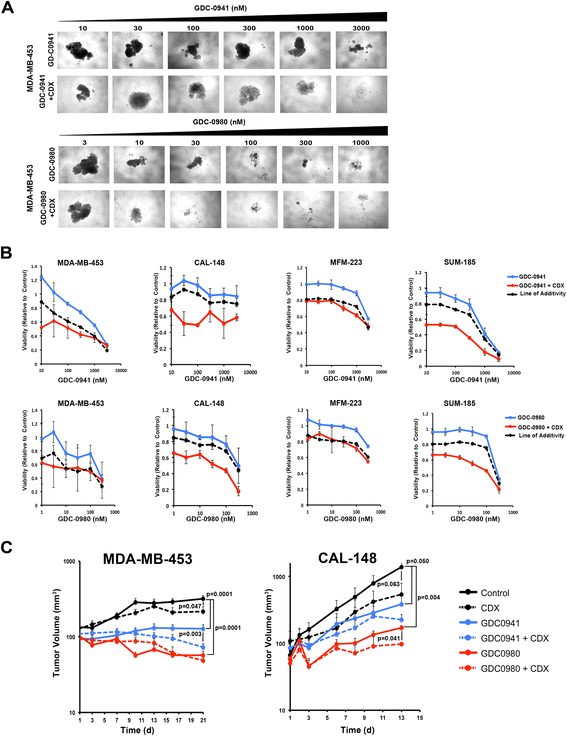

Supplement: Supplementary file 18 — Authors’ original file for figure 6 [file 13058_2014_406_MOESM18_ESM.gif]
