# Supplementary material for: PIK3CA mutations in androgen receptor-positive triple negative breast cancer confer sensitivity to the combination of PI3K and androgen receptor inhibitors
Source: Breast Cancer Res. 2014 Aug 8;16:406. doi: 10.1186/s13058-014-0406-x (PMC4187324; doi:10.1186/s13058-014-0406-x)
Supplement: Supplementary file 8 — Additional file 8: Supplemental methods.(DOCX 101 KB) [file 13058_2014_406_MOESM8_ESM.docx]

**Supplemental Methods**

***Fluorescence-activated cell sorting and PIK3CA sequencing of AR expressing TNBC cell lines.*** AR positive cell lines (1 x 10^6^) were harvested and fixed in 4% paraformaldehyde followed by permeabliization in cold 90% methanol. Cells were incubated with anti-AR antibody (clone# D6F11, Cell Signaling) (1:100) at room temperature (RT) for 1hr. After a PBS wash cells were incubated with goat anti-rabbit Alexaflour 488 antibody (invitrogen) (1:10,000) at RT for 30 min. CAL-148 cells (5E^5^) were sorted into AR^low^ and AR^high^ populations and DNA extracted (DNA easy, Qiagen). PCR was performed on PIK3CA amplicons similar to above (see PIK3CA mutation evaluation) followed by Sanger sequencing.

***Quantification of apoptosis.***

*Caspase 3/7 activity.*

Cell lines were seeded in triplicate in 96-well plates. Media was removed and replaced with media containing vehicle (control) or indicated drugs. After 48 h, apoptosis was determined by measuring luciferase from activated caspase 3/7 after addition of Caspase-Glo reagent (Promega). Relative levels of caspase activity were normalized to viable cell number determined by metabolic reduction of alamarBlue.

*Cell cycle quantification of DNA fragmentation.*

Cells were seeded in 60mm dishes (1 x 10^6^ cells/dish) in appropriate supplemented medium. Twenty-four hours later, cells were incubated for an additional 48h in the presence of CDX (25 μM), GDC-0941 (1 μM), or GDC-0980 (300 nM). All drugs were added individually as well as in combinations that included CDX with either GDC-0941 or GDC-0980. ADR (3 μM) was used as a positive control. Cell medium was collected while adherent cells were harvested and resuspended in 1 ml cold PBS. To fix cells, 0.7 ml cold ethanol (70%) was added dropwise to each tube while vortexing gently. After samples were incubated overnight at 4$^{\circ}C$, they were washed once with 1 ml PBS, and resuspended in 400 μl PI/Triton X-100 staining solution (0.1% (v/v) Triton X-100 (Sigma) in PBS containing 200 ng/ml DNAse-free RNAse A (Sigma) and 40 μg/ml propidium iodide (PI). Cells were incubated at 37$^{\circ}C$ for 15 min and then transferred to ice and protected from light until fluorescence was read by flow cytometer (3-laser Becton Dickinson LSRII).

***Immunofluorescence.*** Cells were fixed (4% paraformaldehyde) and permeablized (90% cold methanol) prior to 1 h blocking in 1% BSA. Cells were incubated with rabbit anti-AR (1:500, Cell Signaling, D6F11) overnight and then washed with PBS and incubated with anti-rabbit Alexa Flour 488 (1:10:000, invitrogen) for 1 h. Cells were then washed and blocked with rabbit Fab (2) fragments (1:30) for 1 h. Following a wash, cells were incubated in rabbit anti-p-AKT (1:50) overnight. Cells were washed and incubated with anti-rabbit Alexa Flour 594 (1:10,000) for 1 h followed by a wash. Cells were cytospun onto slides and mounted in DAPI containing mounting media.
